# Halogen and Hydrogen Bond Motifs in Ionic Cocrystals Derived from 3-Halopyridinium Halogenides and Perfluorinated Iodobenzenes

**DOI:** 10.1021/acs.cgd.1c00755

**Published:** 2021-10-13

**Authors:** Lidija Posavec, Vinko Nemec, Vladimir Stilinović, Dominik Cinčić

**Affiliations:** Department of Chemistry, Faculty of Science, University of Zagreb, Horvatovac 102a, 10000 Zagreb, Croatia

## Abstract

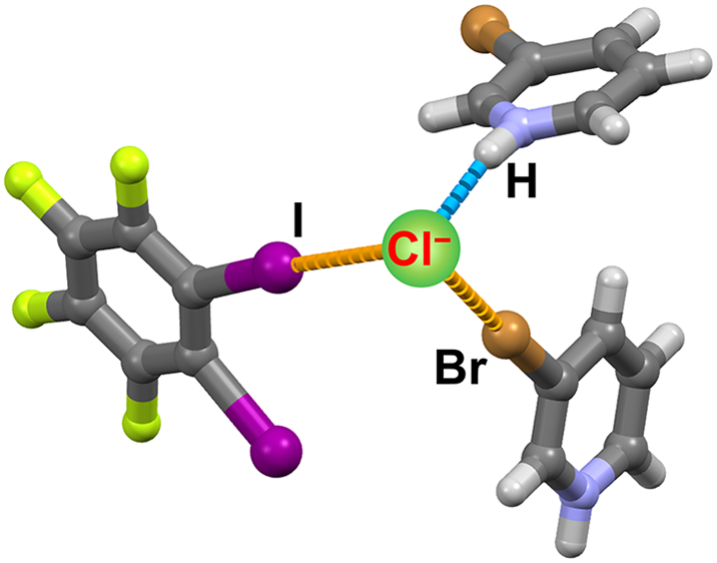

Four halopyridinium salts, 3-chloro- and 3-bromopyridinium chlorides and bromides, have been successfully cocrystallized with two ditopic perfluorinated iodobenzenes, 1,4-diiodotetrafluorobenzene and 1,2-diiodotetrafluorobenzene. These halogen bond donor molecules were chosen because the different positionings of halogen bond donor atoms can lead to different supramolecular architectures. In this work, we present insight into the halogen bond acceptor potential of chloride and bromide ions, as well as the halogen bond donor potential of chlorine and bromine atoms substituted on the pyridinium ring when combined with the expectedly very strong hydrogen bonds between halopyridinium ions and free halogenide anions. A series of eight cocrystals were obtained in which three pairs of isostructural cocrystals were formed. Dominant interactions in the obtained cocrystals were charge-assisted hydrogen bonds between halopyridinium cations and halogenide ions as well as halogen bonds between halogen atoms on the pyridinium ring and halogenide ions.

## Abstract 2

3-Chloro- and 3-bromopyridinium chloride and bromide have been cocrystallized with 1,4-diiodo- and 1,2-diiodotetrafluorobenzene, in order to evaluate both the hydrogen and halogen bond acceptor potential of chloride and bromide ions in combination with the hydrogen and halogen bond donor potential of the halopyridinium cations.

In the last 30 years, crystal engineering has started building on the knowledge obtained from analyzing intermolecular interactions present in crystal structures by moving on to study the design of crystal materials with desired properties and supramolecular topologies.^1−3^ Research into designing multicomponent systems where molecules are linked together using halogen bonds^4^ as dominant intermolecular interactions has been intensifying in the past 20 years.^5−11^ Some of the main advantages of the halogen bonding approach can be found in the exploitation of the nature of the halogen bond,^12−14^ especially regarding its strength and directionality, which can lead to the formation of systems with desired supramolecular architectures.^15−20^ Halogen bond donor strength decreases with a decrease in the radius of the halogen atom,^21,22^ so the majority of systems present in the literature contain iodine donor atoms, while bromine and chlorine atoms are less represented.^23−25^ Anions find widespread use as hydrogen bond acceptors since they are reliable and participate in somewhat predictable interactions. For example, halogenide ions have been studied as hydrogen bond acceptors in simple salts, organic cocrystals, and complex metal–organic systems and also as anion receptors and sensors.^26−28^ They have been proven to be good halogen and hydrogen bond acceptors because of the large charge density.^29−31^ As a result of their sphericity, halogenide ions provide for the possibility of establishing a range of supramolecular interactions with different resultant network geometries and topologies.^32−38^ The number of halogen bonds formed with the anion in the crystal varies and depends on the molecules of which the multicomponent system consists, as well as the geometric requirements of the crystal packing. Halogenide ions typically participate as acceptors of two or three halogen bonds; however, this number can be increased up to eight.^39,40^ In the literature, one can find studies of halogen bonding involving halogenide ions in halopyridinium and haloanilinium salts, cocrystals containing metal complex subunits, cryptated derivatives, quaternary ammonium ions, spiropyran derivatives, etc.^41−47^ While there is a good amount of halogen-bonded structures containing halogenide ions present in the Cambridge Structural Database,^48^ there are few data sets and studies of ionic cocrystals containing perfluorinated iodobenzenes. Most numerous are halogen-bonded ionic cocrystals with 1,3,5-trifluoro-2,4,6-triiodobenzene (35 hits), 1,4-diiodotetrafluorobenzene (29 hits), 1,3-diiodotetrafluorobenzene (9 hits), 1,2-diiodotetrafluorobenzene (7 hits), and iodopentafluorobenzene (1 hit). The studied systems mostly involve networks formed by halogen-bonded donor molecules and halogenide ions which surround organic cations, such as the tetrabuthylammonium cation, or metal−organic complexes.

In this work, we explored the ability of two halogenide ions, chloride and bromide, to act as halogen bond acceptors, and simultaneously the ability of a chlorine or bromine halogen atom located on the halopyridinium cation to act as a halogen bond donor. As coformers, we chose perfluorinated aromatic halogen bond donor molecules: 1,4-diiodotetrafluorobenzene (**14tfib**) and 1,2-diiodotetrafluorobenzene (**12tfib**). These regioisomers were chosen because they are both ditopic donors and also because of their specific geometric features. **14tfib** is usually present in structures as a linear ditopic donor, while **12tfib** exhibits bent ditopic geometry with a 60° angle of propagation. Acceptor coformers were a series of 3-halopyridinium halogenide salts, 3-chloropyridinium chloride (**Clpy**HCl), 3-chloropyridinium bromide (**Clpy**HBr), 3-bromopyridinium chloride (**Brpy**HCl), and 3-bromopyridinium bromide (**Brpy**HBr).

**Scheme 1:**
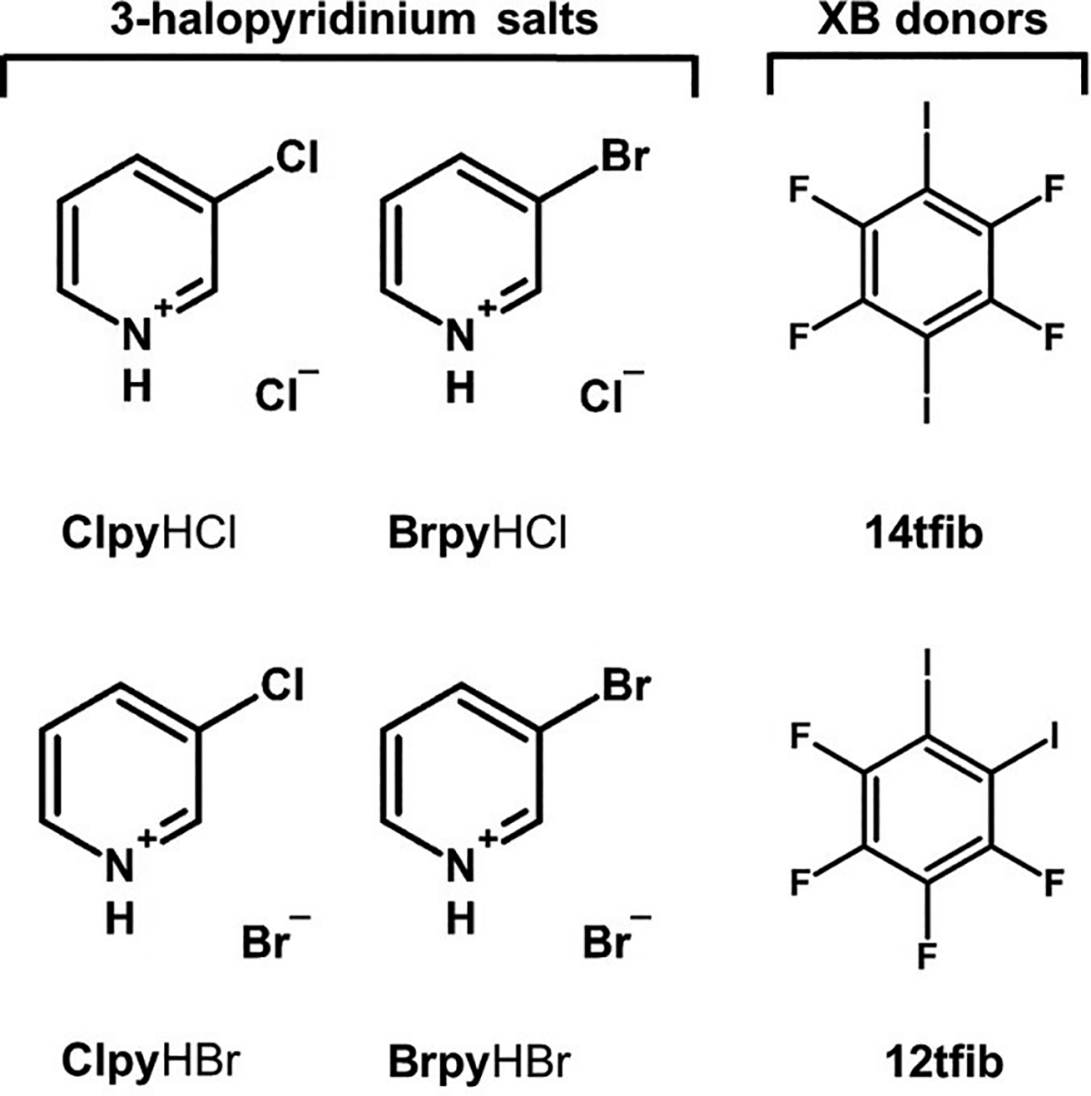
Molecular Structures of 3-Halopyridinium Salts and Halogen Bond Donors, Perhalogenated Benzenes, Used in This Study

Cocrystallization experiments were performed both mechanochemically, using liquid-assisted grinding (LAG)^49,50^ and from the solution. The mechanochemical reactions were performed in a Retsch MM200 ball mill using 10 mL stainless steel jars and one stainless steel ball 12 mm in diameter per jar in order to ensure reaction completion. The resulting products were characterized using powder X-ray diffraction. Crystallization experiments were performed by dissolving reactants in an appropriate solvent or a mixture of solvents and then letting the solvents evaporate at room temperature until a crystalline product was formed (see Supporting Information). The obtained crystal products were characterized by powder X-ray diffraction (PXRD), thermogravimetry (TG), differential scanning calorimetry (DSC), and single crystal X-ray diffraction (SCXRD).

Structural analysis of the synthetized crystals showed that eight new halogen-bonded ionic cocrystals were obtained: (**Clpy**HCl)(**14tfib**), (**Clpy**HCl)_2_(**12tfib**), (**Brpy**HCl)(**14tfib**), (**Brpy**HCl)_2_(**12tfib**), (**Clpy**HBr)_2_(**14tfib**), (**Clpy**HBr)_2_(**12tfib**), (**Brpy**HBr)_2_(**14tfib**), and (**Brpy**HBr)(**12tfib**)_2_ (Figure 1).

**Figure 1:**
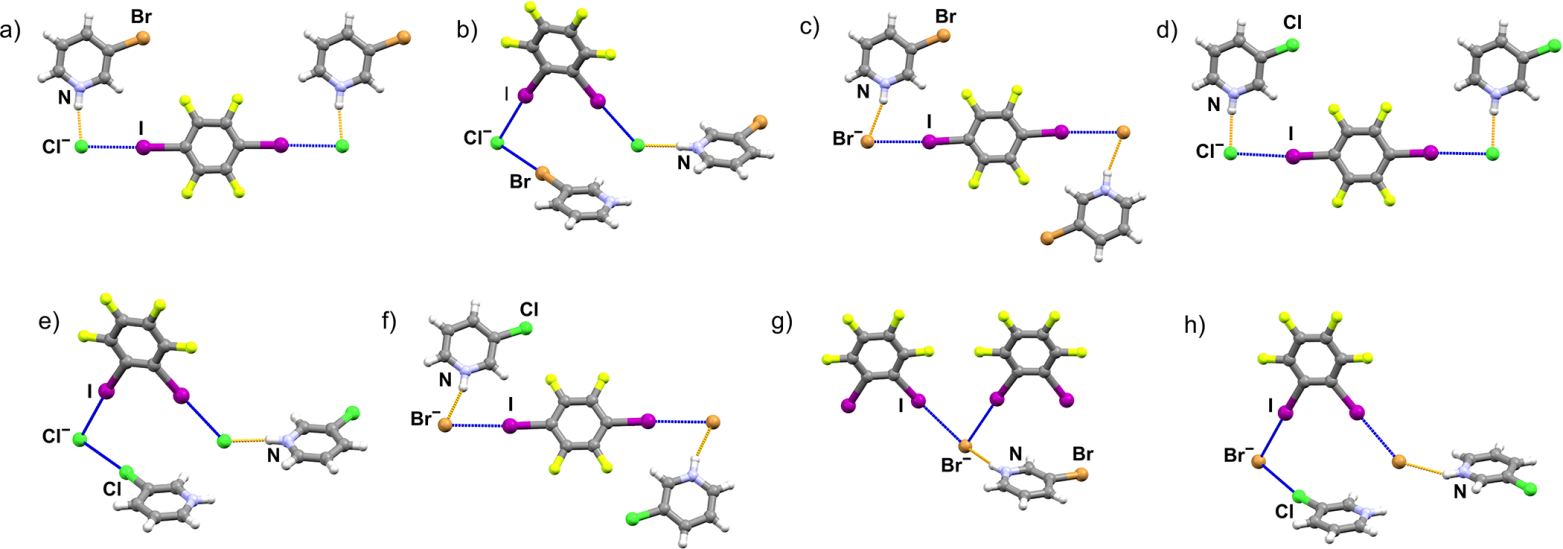
N–H···X^–^ hydrogen bonds (orange) and halogen bonds (blue) in the synthesized cocrystals: (a) (**Brpy**HCl)(**14tfib**), (b) (**Brpy**HCl)_2_(**12tfib**), (c) (**Brpy**HBr)_2_(**14tfib**), (d) (**Clpy**HCl)(**14tfib**), (e) (**Clpy**HCl)_2_(**12tfib**), (f) (**Clpy**HBr)_2_(**14tfib**), (g) (**Brpy**HBr)(**12tfib**)_2_, (h) (**Clpy**HBr)_2_(**12tfib**).

The two series of synthesized ionic cocrystals contain three isostructural pairs: (**Brpy**HCl)(**14tfib**) and (**Clpy**HCl)(**14tfib**), (**Brpy**HBr)_2_(**14tfib**) and (**Clpy**HBr)_2_(**14tfib**), and (**Brpy**HCl)_2_(**12tfib**) and (**Clpy**HCl)_2_(**12tfib**).

In the (**Clpy**HCl)(**14tfib**) cocrystal, halogen bonding is observed between iodine atoms of **14tfib** and chloride ions. Chloride ions function as ditopic halogen bond acceptors, while molecules of **14tfib** behave as ditopic halogen bond donors. In addition to participating in halogen bond formation, each chloride ion also expectedly functions as an acceptor of an N^+^–H···Cl^–^ charge-assisted hydrogen bond. The repetition of these motifs leads to the formation of a planar supramolecular network (Figure 2a). If an analogy with organometallic networks is drawn, chloride ions take on the role of nodes, while **14tfib** molecules and pyridinium cations play the role of linkers. The crystal structure is layered, with a distance of 3.36 Å between the parallel planes defined by atoms in **14tfib** molecules. Although the (**Brpy**HCl)(**14tfib**) cocrystal displays practically an equivalent supramolecular architecture, the halogen bonds present are shorter and closer to 180° (Table 1).

**Figure 2:**
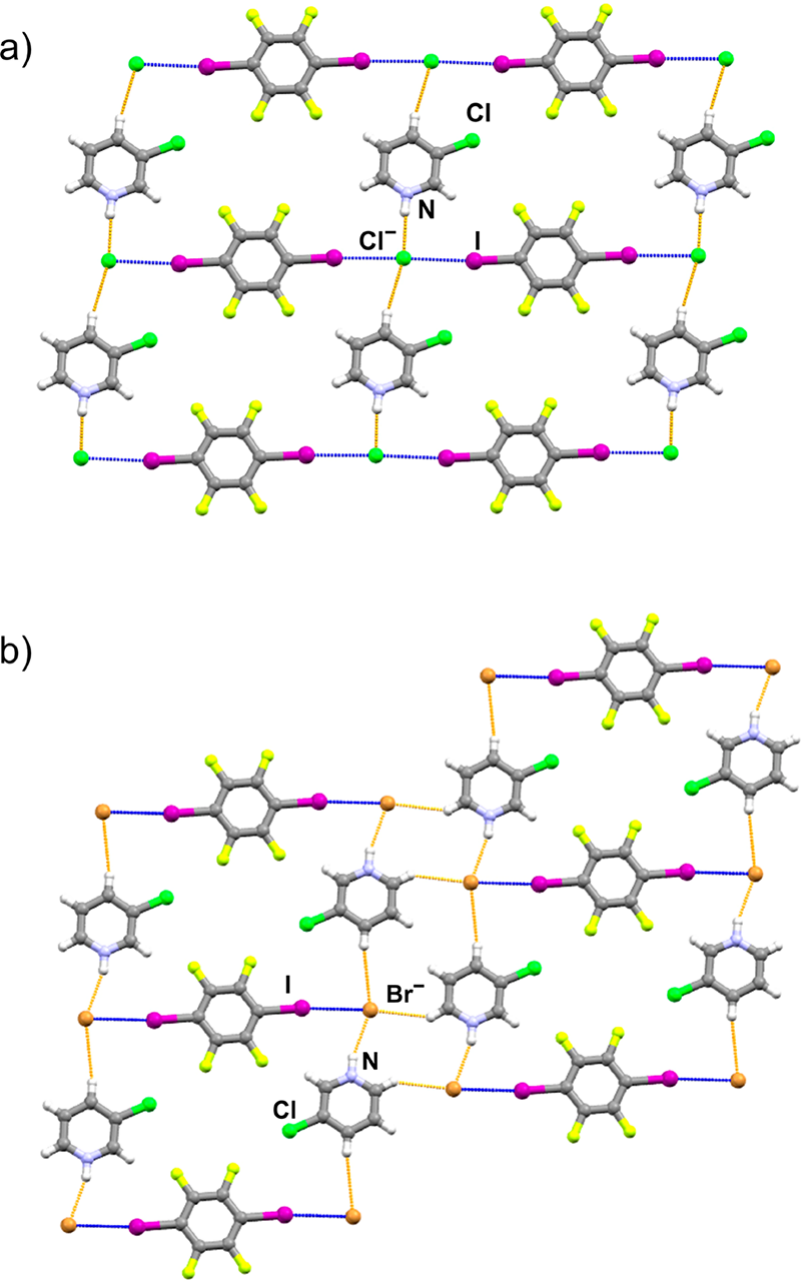
Hydrogen bonds (orange) and halogen bonds (blue) in crystal structures showcasing the halogen- and hydrogen-bonded networks in (a) (**Clpy**HCl)(**14tfib**) (isostructural with (**Brpy**HCl)(**14tfib**)) and (b) (**Clpy**HBr)_2_(**14tfib**) (isostructural with (**Brpy**HBr)_2_(**14tfib**)).

**Table 1:** Halogen Bond Lengths (*d*), Angles (∠), and Relative Shortenings (R.S.) of D···A Distances in the Herein Prepared Cocrystals

cocrystal	D···A	*d*(D···A)/Å	R.S.^a^/%	∠(C–D···A)/°
(**Clpy**HCl)(**14tfib**)	I1···Cl2	3.157	15.4	177.1
	I2···Cl2	3.203	14.1	174.5
(**Clpy**HCl)_2_(**12tfib**)	I2···Cl4	3.214(2)	13.8	175.1
	I1···Cl2	3.194(2)	14.4	178.1
	Cl1···Cl4	3.453(2)	1.3	176.6
(**Brpy**HCl)(**14tfib**)	I1···Cl1	3.167	15.1	179.1
	I2···Cl1	3.125	16.2	178.8
(**Brpy**HCl)_2_(**12tfib**)	I2···Cl2	3.251(3)	12.8	174.2
	I1···Cl1	3.219(4)	13.7	177.1
	Br1···Cl2	3.387(3)	5.9	178.2
(**Clpy**HBr)_2_(**14tfib**)	I1···Br1	3.3082(6)	13.6	172.7
(**Clpy**HBr)_2_(**12tfib**)	I1···Br1	3.3719(6)	12.0	174.7
	I2···Br2	3.3409(5)	12.8	179.1
	Cl2···Br1	3.458(2)	3.9	170.5
(**Brpy**HBr)_2_(**14tfib**)	I1···Br1	3.304(1)	13.7	173.2
(**Brpy**HBr)(**12tfib**)_2_	I1···Br2	3.426(1)	10.5	171.8
	I2···Br2	3.603(1)	5.9	173.3
	I3···Br2	3.377(1)	11.8	176.4
	Br1···Br2	3.830(1)	–4.6	173.4

a R.S. = 1 – *d*(D···A)/[*r*_vdW_(D) + *r*_vdW_(A)].

By exchanging chloride with bromide ions, a significant change in stoichiometry and supramolecular architecture is observed. In the isostructural (**Clpy**HBr)_2_(**14tfib**) and (**Brpy**HBr)_2_(**14tfib**) cocrystals, bromide ions function as monotopic halogen bond acceptors, while **14tfib** molecules behave as ditopic halogen bond donors (Figure 2b). Planar layers are formed, but in this case, each bromide ion participates in only one halogen bond with **14tfib**, one N^+^–H···Cl^–^ charge-assisted hydrogen bond, and two C–H···Cl^–^ contacts.

Combining the bent ditopic halogen bond donor **12tfib** with 3-halopyridinum salts also resulted in the formation of two isostructural ionic cocrystals. The crystal structures of (**Clpy**HCl)_2_(**12tfib**) and (**Brpy**HCl)_2_(**12tfib**) demonstrate the potential of chlorine and bromine atoms to behave as halogen bond donors. In these two crystal structures, two symmetrically inequivalent chloride ions are present. Halogen bonding is observed between the iodine atoms of **12tfib** and chloride ions, and also between the chlorine or bromine atom of the halopyridinium cation and one chloride ion. These cation–anion halogen bonds are relatively weak, which is seen from the fact that contacts Cl···Cl^–^ and Br···Cl^–^ are 1.3% and 5.9% less, respectively, than the sum of the van der Waals radii^51^ (Table 1). These halogen bonds complement the expected N^+^–H···Cl^–^ charge-assisted hydrogen bonds (relative shortening of about 7.6% and 10.2%, Table 2) and additional C–H···Cl^–^ contacts.

**Table 2:** Hydrogen Bond Lengths (*d*), Angles (∠), and Relative Shortenings (R.S.) of D···A Distances in the Herein Prepared Cocrystals

cocrystal	D···A	*d*(D···A)/Å	R.S.^a^/%	∠(C–D···A)/°
(**Clpy**HCl)(**14tfib**)	N1–H1···Cl2	3.037	8.0	174.7
	C3–H3···Cl2	3.203	7.2	174.5
(**Clpy**HCl)_2_(**12tfib**)	N1–H1N···Cl2	3.049(6)	7.6	163.6
	N2–H2N···Cl4	2.965(6)	10.2	166.3
(**Brpy**HCl)(**14tfib**)	N1–H1N···Cl1	3.006	8.9	170.2
(**Brpy**HCl)_2_(**12tfib**)	N1–H1N···Cl1	3.043	7.8	163.5
	N2–H2N···Cl2	2.962	10.2	162.2
(**Clpy**HBr)_2_(**14tfib**)	N1–H1···Br1	3.206(4)	5.7	160.4
(**Clpy**HBr)_2_(**12tfib**)	N1–H1N···Br1	3.154(4)	7.2	168.0
	N2–H2N···Br2	3.308(3)	2.7	145.9
(**Brpy**HBr)_2_(**14tfib**)	N1–H1···Br2	3.236	8.8	157.7
(**Brpy**HBr)(**12tfib**)_2_	N1–H1···Br2	3.216(7)	9.4	161.2

a R.S. = 1 – *d*(D···A)/[*r*_vdW_(D) + *r*_vdW_(A)].

Unlike the other cocrystal pairs, ionic cocrystals containing **12tfib** and the bromide salts are not isostructural. The supramolecular connectivity in (**Clpy**HBr)_2_(**12tfib**) is similar to the ionic cocrystals with **12tfib** and chloride ions. Two symmetrically inequivalent bromide ions are present in the crystal structure. Bromide ions participate as acceptors in a C–I···Br^–^ halogen bond with **12tfib**, and also in one N^+^–H···Br^–^ hydrogen bond and two C–H···Br^–^ contacts with halopyridinium cations. Similarities extend to the crystal packing, the main difference being in the orientation of chloropyridinium cations, which then form different networks around donor molecules than those in halopyridinium chloride cocrystals (comparison on Figure 3a,b). The (**Brpy**HBr)(**12tfib**)_2_ cocrystal differs from all other cocrystals not only in stoichiometry but also in intramolecular connectivity. Each bromide ion acts as a tritopic hydrogen bond and tetratopic halogen bond acceptor, while the two symmetrically inequivalent **12tfib** molecules display both monotopic and ditopic behavior. According to the normally used Bondi’s van der Waals radii, the C–Br···Br^–^ contacts between halopyridinium cations and bromide anions are 4.6% longer than the sum of the contact atoms’ van der Waals radii which would indicate that the bromide ion is an acceptor of only three I···Br^–^ contacts. However, if one is to consider the recent reevaluation of the bromine van der Waals radius put forward by Chernyshov (2.00 Å),^52^ as well as the geometry of the contact (∠ (C–Br···Br^–^) = 173.4°), then this contact can be considered a (relatively weak) halogen bond (relative shortening of 4.3% on the Chernyshov scale), making the bromide anion a tetratopic halogen bond acceptor. Layers are formed by hydrogen bonding and contacts between 3-bromopyridinium cations and bromide anions (Figure 3c). Halogen bond donor molecules are placed at an angle toward these layers, and this combination of halogen and hydrogen bonding and hydrogen contacts results in the formation of a three-dimensional supramolecular network.

**Figure 3:**
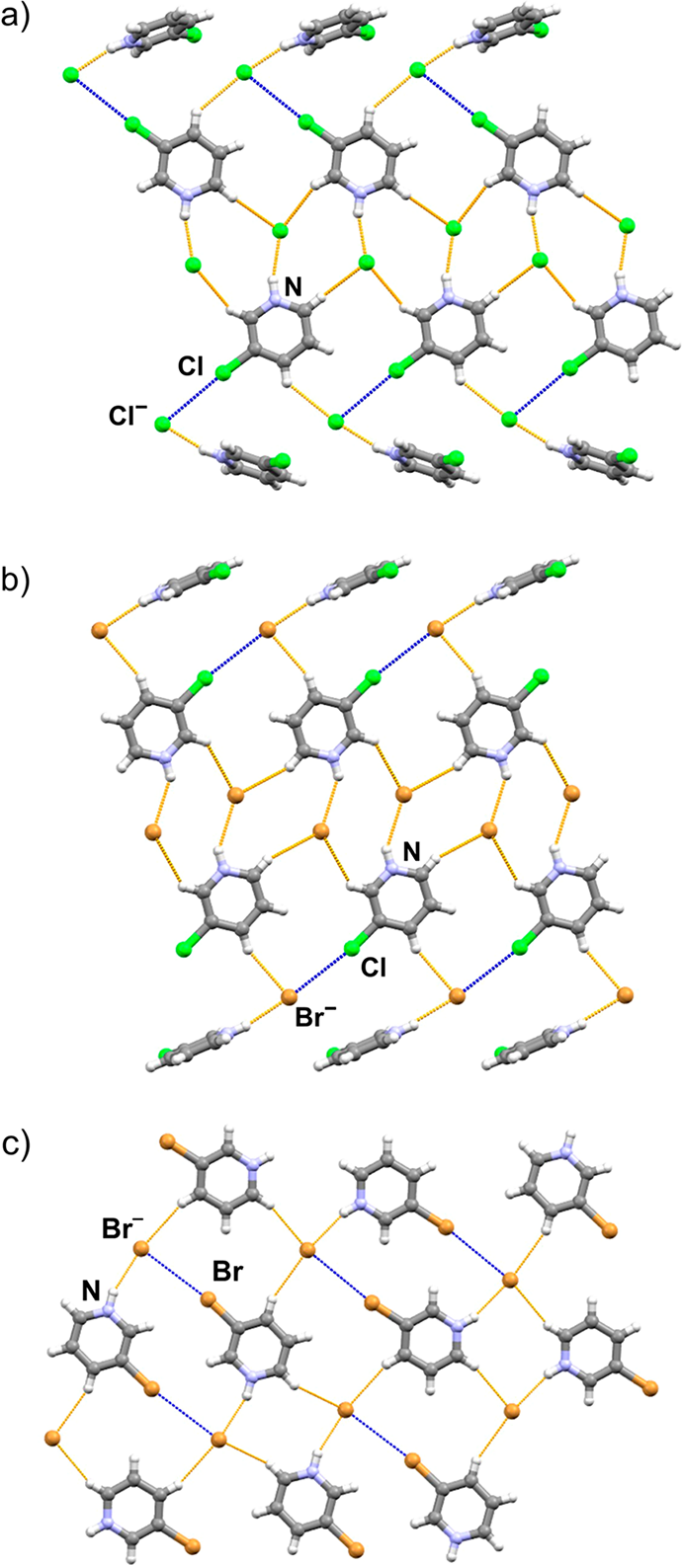
Hydrogen bonds and contacts (orange) and halogen bonds (blue) in the crystal structures showcasing parts of the (a) halogen- and hydrogen-bonded network in (**Clpy**HCl)_2_(**12tfib**), (b) halogen- and hydrogen-bonded network in (**Clpy**HBr)_2_(**12tfib**), and (c) hydrogen-bonded network in (**Brpy**HBr)(**12tfib**)_2_.

A comparison of supramolecular connectivity and packing in the pure salts, (**Clpy**HCl),^31^ (**Clpy**HBr),^32^ (**Brpy**HCl),^32^ and (**Brpy**HBr),^31^ with the cocrystals obtained in this work reveals the following: (**Clpy**HCl) and (**Brpy**HCl) retain discrete tetramer structures of three 3-halopyridinium cations and one halogenide anion only in cocrystals with the weaker donor, **12tfib**. In cocrystals with **14tfib**, only N^+^–H···Cl^–^ hydrogen bonds are retained, while the architecture established by halogen bonding also leads to the straightening of one C–H···Cl^–^ contact from its previous out-of-plane position. Pure (**Brpy**HBr) features a complex structure which can be described as a combination of hydrogen-bonded ribbon-like chains. Again, **14tfib** as the stronger halogen bond donor has a more significant effect on this motif since only a hydrogen-bonded tetramer fragment comprising two 3-bromopyridinium cations and two bromide anions is retained in the cocrystal, while in the **12tfib** cocrystal, the ribbon-like chain motif is modified into a 2D layer with the perpendicular hydrogen bonding contacts to neighboring (**Brpy**HBr) ion pairs substituted by halogen bonds with **12tfib** molecules. In the case of (**Clpy**HBr), contrary to the previous systems, the ribbon-like chain of the pure coformer is retained in the (**Clpy**HBr)_2_(**14tfib**) cocrystal, with the motif extended into a layer by the addition of halogen bonding, while in the cocrystal with **12tfib** only one of the symmetrically inequivalent bromide ions retains a similar hydrogen-bonded tetramer, where the nearby fourth hydrogen contact is changed through the repositioning of the adjacent 3-chloropyridinium cation, as well as by the addition of halogen bonding.

Thermal analysis results (collected in Table 3) expectedly show that all salt cocrystals have higher decomposition temperatures than melting points of the pure halogen bond donors, but no simple trend can be found in a comparison with the melting points of pure salts. Cocrystal decomposition temperatures are mostly lower than salt melting points, with the exception of the isostructural pair, (**Clpy**HBr)_2_(**14tfib**) and (**Brpy**HBr)_2_(**14tfib**), and the (**Brpy**HBr)(**12tfib**)_2_ cocrystal. This trend is similar to the one determined for melting points of two-component cocrystals that most systems have melting points in between the melting points of the coformers used.^53,54^ It is, however, interesting to note that bromide salt cocrystals have higher decomposition temperatures than chloride salt cocrystals.

**Table 3:** Melting and Decomposition Onset Temperatures Determined by the DSC Method for the Reactants and Products in This Work

compound	onset temperature/°C
**14tfib**	110.0^a^
**12tfib**	50.0^a^
**ClpyHCl**	138.2^b^
**BrpyHCl**	160.6^b^
**ClpyHBr**	152.2^b^
**BrpyHBr**	127.1^b^
**(ClpyHCl)(14tfib)**	126.2^b^
**(BrpyHCl)(14tfib)**	114.9^b^
**(ClpyHBr)**_**2**_**(14tfib)**	174.5^b^
**(BrpyHBr)**_**2**_**(14tfib)**	162.5^b^
**(ClpyHCl)**_**2**_**(12tfib)**	110.0^b^
**(BrpyHCl)**_**2**_**(12tfib)**	127.0^b^
**(ClpyHBr)**_**2**_**(12tfib)**	132.9^b^
**(BrpyHBr)(12tfib)**_**2**_	181.5^b^

a Melting point.

b Decomposition temperature.

To conclude, out of the eight obtained ionic cocrystals, six comprise three isostructural cocrystal pairs. In keeping with the hypothesis, the dominant supramolecular interactions in the cocrystals are the N^+^–H···X^–^ hydrogen bonds and I···X^–^ halogen bonds, with additional stabilization afforded by C–H···X^–^ hydrogen bonds and contacts (where X^–^ = Br^–^ or Cl^–^), as well as by halogen bonding through halogen atoms located on the halopyridinium cation. Furthermore, halogen bonding with the selected donor molecules proved largely capable of interfering with the supramolecular motifs present in the pure halopyridinium halogenides. In cocrystals containing **14tfib**, a linear ditopic donor, two-dimensional networks are formed (Figure 4a,b), while on the other hand, in cocrystals containing **12tfib**, a bent ditopic donor, three-dimensional networks are formed, which can be considered as two-dimensional networks (Figure 3) connected by N^+^–H···X^–^ and C–H···X^–^ hydrogen bonds and secondary Y···X^–^ halogen bonds (where X^–^ = Br^–^ or Cl^–^, and Y = halopyridinium Br or Cl atom) that are further connected by I···X^–^ halogen bonds into a three-dimensional network (Figure 4c–e). Contrary to expectations, the presence of secondary halogen bonding is dependent more on the overall crystal packing than on the donor strength of the halopyridinium cations used, as evidenced by the fact that these halogen bonds are present only in three crystal structures and that they can be considered weak according to the relative shortening parameters of 1–6% (especially when compared to the I···X^–^ halogen bond with relative shortening of >10%).

**Figure 4:**
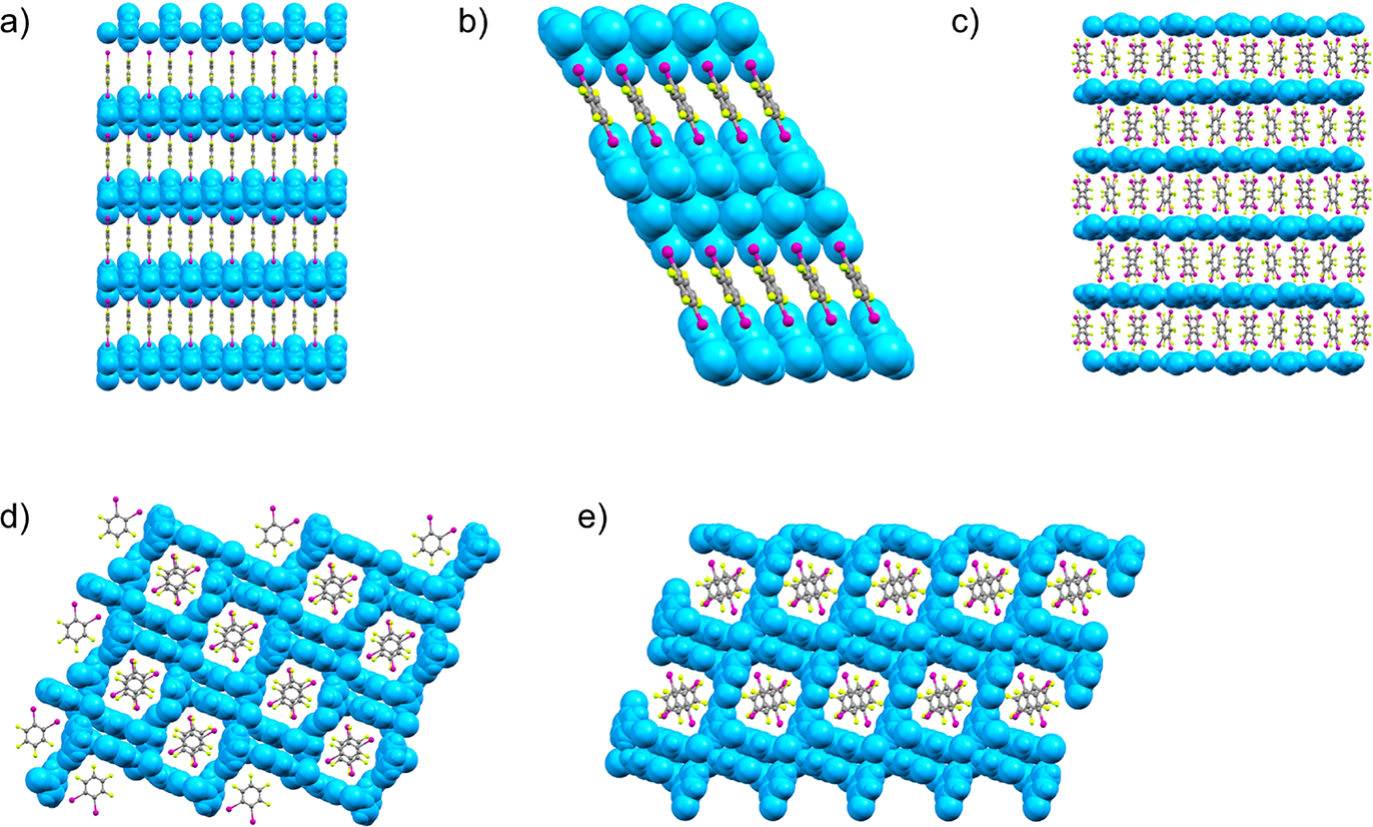
Fragments of the crystal structures showcasing molecular packing in cocrystals of (a) (**Clpy**HCl)(**14tfib**) and (**Brpy**HCl)(**14tfib**), (b) (**Clpy**HBr)_2_(**14tfib**) and (**Brpy**HBr)_2_(**14tfib**), (c) (**Brpy**HBr)(**12tfib**)_2_, (d) (**Clpy**HCl)_2_(**12tfib**) and (**Brpy**HCl)_2_(**12tfib**), (e) (**Clpy**HBr)_2_(**12tfib**).

## Supporting Information Available

Experimental details, including DSC curves, PXRD patterns and single crystal diffraction data (PDF)

## Accession Codes

CCDC 2092847–2092854 contain the supplementary crystallographic data for this paper. These data can be obtained free of charge via www.ccdc.cam.ac.uk/data_request/cif, or by emailing data_request@ccdc.cam.ac.uk, or by contacting The Cambridge Crystallographic Data Centre, 12 Union Road, Cambridge CB2 1EZ, UK; fax: +44 1223 336033.
