# Supplementary material for: Halogen and Hydrogen Bond Motifs in Ionic Cocrystals Derived from 3-Halopyridinium Halogenides and Perfluorinated Iodobenzenes
Source: Cryst Growth Des. 2021 Oct 13;21(11):6044–50. doi: 10.1021/acs.cgd.1c00755 (PMC8569900; doi:10.1021/acs.cgd.1c00755)
Supplement: Supplementary file 1 — cg1c00755_si_001.pdf [file cg1c00755_si_001.pdf]

SUPPORTING INFORMATION

Halogen and hydrogen bond motifs in ionic cocrystals derived from 3-halopyridinium halogenides and perfluorinated iodobenzenes

Lidija Posavec, Vinko Nemec, Vladimir Stilinović* and Dominik Cinčić*

Department of Chemistry, Faculty of Science, University of Zagreb, Horvatovac 102a, HR-10000 Zagreb, Croatia

Email: dominik@chem.pmf.hr, vstilinovic@chem.pmf.hr
Fax: +385 1 4606 341
Tel: +385 1 4606 362

Table of Contents

Experimental details	Mechanochemical syntheses, solution syntheses, thermal analysis, powder X-ray diffraction experiments, single crystal X-ray diffraction experiments	3
Table S1.	Crystal data and refinement details for the prepared cocrystals.	8
Figure S1.	Molecular structure of (**Clpy**HCl)(**14tfib**) showing the atom-labeling scheme. Displacement ellipsoids are drawn at the 50 % probability level, and H atoms are shown as small spheres of arbitrary radius.	12
Figure S2.	Molecular structure of $(\textbf{Clpy}\text{HCl})_2$(**12tfib**) showing the atom-labeling scheme. Displacement ellipsoids are drawn at the 50 % probability level, and H atoms are shown as small spheres of arbitrary radius.	12
Figure S3.	Molecular structure of $(\textbf{Clpy}\text{HBr})_2$(**14tfib**) showing the atom-labeling scheme. Displacement ellipsoids are drawn at the 50 % probability level, and H atoms are shown as small spheres of arbitrary radius.	13
Figure S4.	Molecular structure of $(\textbf{Clpy}\text{HBr})_2$(**12tfib**) showing the atom-labeling scheme. Displacement ellipsoids are drawn at the 50 % probability level, and H atoms are shown as small spheres of arbitrary radius.	13
Figure S5.	Molecular structure of (**Brpy**HCl)(**14tfib**) showing the atom-labeling scheme. Displacement ellipsoids are drawn at the 50 % probability level, and H atoms are shown as small spheres of arbitrary radius.	14
Figure S6.	Molecular structure of $(\textbf{Brpy}\text{HCl})_2$(**12tfib**) showing the atom-labeling scheme. Displacement ellipsoids are drawn at the 50 % probability level, and H atoms are shown as small spheres of arbitrary radius.	14
Figure S7.	Molecular structure of $(\textbf{Brpy}\text{HBr})_2$(**14tfib**) showing the atom-labeling scheme. Displacement ellipsoids are drawn at the 50 % probability level, and H atoms are shown as small spheres of arbitrary radius.	15
Figure S8.	Molecular structure of (**Brpy**HBr)$(\textbf{12tfib})_2$ showing the atom-labeling scheme. Displacement ellipsoids are drawn at the 50 % probability level, and H atoms are shown as small spheres of arbitrary radius.	15
Figure S9.	Comparison of powder patterns generated from single crystal data for isostructural cocrystals.	16
Figure S10.	PXRD pattern of **14tfib**.	16
Figure S11.	PXRD pattern of **12tfib**.	17
Figure S12.	PXRD patterns of: a) **14tfib**, b) **Clpy**HCl, c) product obtained by grinding **14tfib** and **Clpy**HCl in a 1:1 stoichiometric ratio, d) calculated pattern from (**Clpy**HCl)(**14tfib**) single crystal data.	17

Figure S13.	PXRD patterns of: a) **12tfib**, b) **Clpy**HCl, c) product obtained by grinding **12tfib** and **Clpy**HCl in a 1:2 stoichiometric ratio d) calculated pattern from (**Clpy**HCl)$_2$(**12tfib**) single crystal data.	18
Figure S14.	PXRD patterns of: a) **14tfib**, b) **Brpy**HCl, c) product obtained by grinding **14tfib** and **Brpy**HCl in a 1:1 stoichiometric ratio, d) calculated pattern from (**Brpy**HCl)(**14tfib**) single crystal data.	18
Figure S15.	PXRD patterns of: a) **12tfib**, b) **Brpy**HCl, c) product obtained by grinding **12tfib** and **Brpy**HCl in a 1:2 stoichiometric ratio, d) calculated pattern from (**Brpy**HCl)$_2$(**12tfib**) single crystal data.	19
Figure S16.	PXRD patterns of: a) **14tfib**, b)**Clpy**HBr, c) product obtained by grinding **14tfib** and **Clpy**HBr in a 1:2 stoichiometric ratio, d) calculated pattern from (**Clpy**HBr)$_2$(**14tfib**) single crystal data.	19
Figure S17.	PXRD patterns of: a) **12tfib**, b) **Clpy**HBr, c) product obtained by grinding **12tfib** and **Clpy**HBr in a 1:2 stoichiometric ratio, d) calculated pattern from (**Clpy**HBr)$_2$(**12tfib**) single crystal data.	20
Figure S18.	PXRD patterns of: a) **14tfib**, b) **Brpy**HBr, c) product obtained by grinding **14tfib** and **Brpy**HBr in a 1:2 stoichiometric ratio, d) calculated pattern from (**Brpy**HBr)$_2$(**14tfib**) single crystal data.	20
Figure S19.	PXRD patterns of: a) **12tfib**, b) **Brpy**HBr, c) product obtained by grinding **12tfib** and **Brpy**HBr in a 2:1 stoichiometric ratio, d) calculated pattern from (**Brpy**HBr)(**12tfib**)$_2$ single crystal data.	21
Figure S20.	DSC curve of **12tfib**.	21
Figure S21.	DSC curve of **14tfib**.	22
Figure S22.	DSC curve of **Clpy**HCl.	22
Figure S23.	DSC curve of **Brpy**HCl.	23
Figure S24.	DSC curve of **Clpy**HBr.	23
Figure S25.	DSC curve of **Brpy**HBr.	24
Figure S26.	TG and DSC curves of (**Clpy**HCl)(**14tfib**).	24
Figure S27.	Blank-corrected DSC curve of (**Clpy**HCl)(**14tfib**).	25
Figure S28.	TG and DSC curves of (**Clpy**HCl)(**12tfib**).	25
Figure S29.	Blank-corrected DSC curve of (**Clpy**HCl)(**12tfib**).	26
Figure S30.	TG and DSC curves of (**Brpy**HCl)(**14tfib**).	26
Figure S31.	Blank-corrected DSC curve of (**Brpy**HCl)(**14tfib**).	27
Figure S32.	TG and DSC curves of (**Brpy**HCl)$_2$(**12tfib**).	27
Figure S33.	Blank-corrected DSC curve of (**Brpy**HCl)$_2$(**12tfib**).	28
Figure S34.	TG and DSC curves of (**Clpy**HBr)$_2$(**14tfib**).	28
Figure S35.	Blank-corrected DSC curve of (**Clpy**HBr)$_2$(**14tfib**).	29
Figure S36.	TG and DSC curves of (**Clpy**HBr)$_2$(**12tfib**).	29
Figure S37.	Blank-corrected DSC curve of (**Clpy**HBr)$_2$(**12tfib**).	30
Figure S38.	TG and DSC curves of (**Brpy**HBr)$_2$(**14tfib**).	30
Figure S39.	Blank-corrected DSC curve of (**Brpy**HBr)$_2$(**14tfib**).	31
Figure S40.	TG and DSC curves of (**Brpy**HBr)(**12tfib**)$_2$.	31
Figure S41.	Blank-corrected DSC curve of (**Brpy**HBr)(**12tfib**)$_2$.	32

EXPERIMENTAL DETAILS

Preparation of **Clpy**HCl

0.25 mL (2.6 mmol) of 3-chloropyridine was added in 2 mL of acetonitrile. In prepared solution then was added 80 mL of concentrated hydrochloric acid and left to crystallize at room temperature for couple of days.

Preparation of **Clpy**HBr

0.25 mL (2.6 mmol) of 3-chloropyridine was added in 2 mL of acetonitrile. In prepared solution than was added 80 mL of concentrated hydrobromic acid and left to crystallize at room temperature for couple of days.

Preparation of **Brpy**HCl

0.25 mL (2.6 mmol) of 3-bromopyridine was added in 2 mL of acetonitrile. In prepared solution than was added 80 mL of concentrated hydrochloric acid and left to crystallize at room temperature for couple of days.

Preparation of **Brpy**HBr

0.25 mL (2.6 mmol) of 3-bromopyridine was added in 2 mL of acetonitrile. In prepared solution than was added 80 mL of concentrated hydrobromic acid and left to crystallize at room temperature for couple of days.

MECHANOCHEMICAL SYNTHESES

Synthesis of (**Clpy**HCl)(**14tfib**)

A mixture of **Clpy**HCl (40.8 mg, 272 μmol) and **14tfib** (109.2 mg, 272 μmol) was placed in a 10 mL stainless steel jar along with 20 μL nitromethane and one stainless steel ball 12 mm in diameter. The reaction mixture was then milled for 30 minutes in a Retsch MM200 Shaker Mill operating at 25 Hz.

Synthesis of (**Clpy**HCl)$_2$(**12tfib**)

A mixture of **Clpy**HCl (64.1 mg, 427 μmol) and **12tfib** (85.9 mg, 214 μmol) was placed in a 10 mL stainless steel jar along with 20 μL nitromethane and one stainless steel ball 12 mm in diameter. The reaction mixture was then milled for 30 minutes in a Retsch MM200 Shaker Mill operating at 25 Hz.

Synthesis of (**Brpy**HCl)(**14tfib**)

A mixture of **Brpy**HCl (48.9 mg, 251 μmol) and **14tfib** (101.1 mg, 251 μmol) was placed in a 10 mL stainless steel jar along with 20 μL nitromethane and one stainless steel ball 12 mm in diameter. The reaction mixture was then milled for 30 minutes in a Retsch MM200 Shaker Mill operating at 25 Hz.

Synthesis of (**Brpy**HCl)$_2$(**12tfib**)

A mixture of **Brpy**HCl (73.8 mg, 379 μmol) and **12tfib** (76.2 mg, 190 μmol) was placed in a 10 mL stainless steel jar along with 20 μL nitromethane and one stainless steel ball 12 mm in diameter. The reaction mixture was then milled for 30 minutes in a Retsch MM200 Shaker Mill operating at 25 Hz.

Synthesis of (**Clpy**HBr)$_2$(**14tfib**)

A mixture of **Clpy**HBr (73.8 mg, 379 μmol) and **14tfib** (76.2 mg, 190 μmol) was placed in a 10 mL stainless steel jar along with 20 μL nitromethane and one stainless steel ball 12 mm in diameter. The reaction mixture was then milled for 30 minutes in a Retsch MM200 Shaker Mill operating at 25 Hz.

Synthesis of (**Clpy**HBr)$_2$(**12tfib**)

A mixture of **Clpy**HBr (73.8 mg, 380 μmol) and **12tfib** (76.2 mg, 190 μmol) was placed in a 10 mL stainless steel jar along with 20 μL nitromethane and one stainless steel ball 12 mm in diameter. The reaction mixture was then milled for 30 minutes in a Retsch MM200 Shaker Mill operating at 25 Hz.

Synthesis of (**Brpy**HBr)$_2$(**14tfib**)

A mixture of **Brpy**HBr (81.5 mg, 341 μmol) and **14tfib** (68.5 mg, 171 μmol) was placed in a 10 mL stainless steel jar along with 20 μL nitromethane and one stainless steel ball 12 mm in diameter. The reaction mixture was then milled for 30 minutes in a Retsch MM200 Shaker Mill operating at 25 Hz.

Synthesis of (**Brpy**HBr)(**12tfib**)$_2$

A mixture of **Brpy**HBr (34.4 mg, 144 μmol) and **12tfib** (115.6 mg, 288 μmol) was placed in a 10 mL stainless steel jar along with 20 μL nitromethane and one stainless steel ball 12 mm in diameter. The reaction mixture was then milled for 30 minutes in a Retsch MM200 Shaker Mill operating at 25 Hz.

SOLUTION SYNTHESES

Single crystals of (**Clpy**HCl)(**14tfib**)

A mixture of **Clpy**HCl (19.4 mg, 129.33 µmol) and **14tfib** (10 mg, 24.9 µmol) was dissolved in 2.0 mL of hot dichloromethane and left to crystallize at room temperature.

Single crystals of $(\textbf{Clpy}\text{HCl})_2$(**12tfib**)

A mixture of **Clpy**HCl (14.8 mg, 98.7 µmol) and **12tfib** (10 mg, 24.9 µmol) was dissolved in 2.0 mL of hot dichloromethane and left to crystallize at room temperature.

Single crystals of (**Brpy**HCl)(**14tfib**)

A mixture of **Brpy**HCl (19.4 mg, 99.8 µmol) and **14tfib** (10 mg, 24.9 µmol) was dissolved in 2.0 mL of hot dichloromethane and left to crystallize at room temperature.

Single crystals of $(\textbf{Brpy}\text{HCl})_2$(**12tfib**)

A mixture of **BrpyHCl** (14.8 mg, 76.1 µmol) and **12tfib** (10 mg, 24.9 µmol) was dissolved in 2.0 mL of hot dichloromethane and left to crystallize at room temperature.

Single crystals of $(\textbf{Clpy}\text{HBr})_2$(**14tfib**)

A mixture of **Clpy**HBr (14.8 mg, 76.1 µmol) and **14tfib** (10 mg, 24.9 µmol) was dissolved in 1.0 mL of hot nitromethane. After dissolving, 1.0 mL of acetonitrile was added into the solution and left to crystallize at room temperature.

Single crystals of $(\textbf{Clpy}\text{HBr})_2$(**12tfib**)

A mixture of **Clpy**HBr (14.8 mg, 99.1 µmol) and **12tfib** (10 mg, 24.9 µmol) was dissolved in 1.0 mL of hot nitromethane. After dissolving, 1.0 mL of diethyl ether was added into the solution and left to crystallize at room temperature and left to crystallize at room temperature.

Single crystals of $(\textbf{Brpy}\text{HBr})_2$(**14tfib**)

A mixture of **Brpy**HBr (23.7 mg, 99.2 µmol) and **14tfib** (20 mg, 49.8 µmol) was dissolved in 1.0 mL of hot nitromethane. After dissolving, 1.0 mL of diethyl ether was added into the solution and left to crystallize at room temperature and left to crystallize at room temperature.

Single crystals of (**Brpy**HBr)(**12tfib**)$_2$

A mixture of **Clpy**HBr (23.7 mg, 99.2 μmol) and **12tfib** (20 mg, 49.8 μmol) was dissolved in 1.0 mL of hot nitromethane. After dissolving, 1.0 mL of diethyl ether was added into the solution and left to crystallize at room temperature and left to crystallize at room temperature.

THERMAL ANALYSIS

TG and DSC measurements were performed on a Mettler-Toledo DSC823^e module and on a Mettler-Toledo TGA/DSC 3+ instrument. The samples were placed in sealed aluminium pans (40 μL) with two pinholes made on the top cover, and heated in flowing nitrogen (50 mL min^{-1}) from 25 °C to 500 °C at a rate of 10 °C min^{-1}. The data collection and analysis was performed using the program package STAR^e Software 15.00.[1]

POWDER X-RAY DIFFRACTION EXPERIMENTS

PXRD experiments were performed on a Malvern PANalytical X-ray diffractometer with Cu*K*α1 (1.54056 Å) radiation at 15 mA and 40 kV. The scattered intensities were measured with a scintillation counter. The angular range was from 5 to 40° (2θ) with steps of 0.02 – 0.03°, and the measuring time was 0.2 – 0.5 s per step. Data collection and analysis was performed using the program package Data Viewer.[2]

SINGLE-CRYSTAL X-RAY DIFFRACTION EXPERIMENTS

The crystal and molecular structures of the prepared cocrystals were determined by single crystal X-ray diffraction. Details of data collection and crystal structure refinement are listed in Table S1, S2, S3 and S4. Diffraction measurements were made on an Oxford Diffraction Xcalibur Kappa CCD X-ray diffractometer and Rigaku Synergy XtaLAB X-ray diffractometer with graphite-monochromated MoKα (λ = 0.71073Å) radiation. The data sets were collected using the ω scan mode over the 2θ range up to 54° (Xcalibur Kappa CCD) and up to 64° (Synergy XtaLAB). Programs CrysAlis CCD, CrysAlis RED and CrysAlisPro were employed for data collection, cell refinement, and data reduction.[3,4] The structures were solved by direct methods and refined using the SHELXS, SHELXT, and SHELXL programs, respectively.[5,6] The structural refinement was performed on F^2 using all data. Hydrogen atoms were placed in calculated positions and treated as riding on their parent atoms. Single crystal diffraction data for (**Clpy**HCl)(**14tfib**), (**Brpy**HCl)(**14tfib**) and (**Clpy**HBr)$_2$(**14tfib**) salt cocrystals was treated during data reduction as resulting from a combination of two twin components. All calculations were performed using the WINGX crystallographic suite of

programs.[7] The molecular structures of compounds and their molecular packing projections were prepared by Mercury.[8]

References

1. STARe Evaluation Software Version 15.00, Mettler–Toledo GmbH, 2016.
2. Data Viewer Version 1.9a, PANalytical B.V. Amelo, The Netherlands, 2018.
3. Oxford Diffraction, Oxford Diffraction Ltd., Xcalibur CCD system, CrysAlis CCD and CrysAlis RED software, Version 1.170, 2003.
4. Rigaku Oxford Diffraction, Gemini CCD system , CrysAlis Pro software, Version 171.41.93a, 2020.
5. (a) G. M. Sheldrick, *Acta Cryst. A*, 2008, **64**, 112–122; (b) G. M. Sheldrick, *Acta Cryst. C*, 2015, **71**, 3–8.
6. G. M. Sheldrick, *Acta Cryst. A*, 2015, **71**, 3–8.
7. L. J. Farrugia, *J. Appl. Cryst.*, 2012, **45**, 849–854.
8. C. F. Macrae, I. J. Bruno, J. A. Chisholm, P. R. Edgington, P. McCabe, E. Pidcock, L. Rodriguez-Monge, R. Taylor, J. v. d. Streek and P. A. Wood, *J. Appl. Crystallogr.* **2008**, 41, 466.

Table S1. Crystal data and refinement details for the prepared compounds.

	(**Clpy**HCl)(**14tfib**)	(**Clpy**HCl)$_2$(**12tfib**)
Molecular formula	$(C_5H_5NCl_2)_2(C_6F_4I_2)$	$(C_5H_5NCl_2)_2(C_6F_4I_2)$
M_r	551.86	701.86
Crystal system	monoclinic	monoclinic
Space group	$P\,2_1/m$	$P\,2_1/n$
Crystal data:		
a / Å	9.3335(19)	14.9352(11)
b / Å	6.7193(17)	7.5337(5)
c / Å	13.356(3)	20.8733(14)
α / °	90	90
β / °	98.759(19)	108.179(8)
γ / °	90	90
V / Å^3	827.9(3)	2231.4(3)
Z	2	4
D_{calc} / g cm^{-3}	2.214	2.089
λ(MoK_α) / Å	0.71073	0.71073
T / K	295	295
Crystal size / mm^3	0.40 x 0.15 x 0.03	0.48 x 0.10 x 0.09
μ / mm^{-1}	4.148	3.336
F(000)	508	1320
Refl. collected/unique	3285 / 1896	16209 / 4848
Parameters/restraints	122 / 0	254 / 0
$\Delta\rho_{max}$, $\Delta\rho_{min}$ / e Å^{-3}	1.260; –0.895	0.693; –0.500
$R[F^2 > 4\sigma(F^2)]$	0.0526	0.0405
w$R(F^2)$	0.1291	0.0791
Goodness-of-fit, S	1.211	0.987

Table S1. continued

	(**Brpy**HCl)(**14tfib**)	(**Brpy**HCl)$_2$(**12tfib**)
Molecular formula	$(C_5H_5NBrCl)_2(C_6F_4I_2)$	$(C_5H_5NBrCl)_2(C_6F_4I_2)$
M_r	596.32	790.78
Crystal system	monoclinic	monoclinic
Space group	$P\ 2_1/m$	$P\ 2_1/n$
Crystal data:		
a / Å	9.2684(15)	15.165(4)
b / Å	6.6064(15)	7.5777(16)
c / Å	13.2938(18)	21.105(6)
α / °	90	90
β / °	97.784(13)	108.75(3)
γ / °	90	90
V / Å^3	806.5(3)	2296.6(11)
Z	2	4
D_{calc} / g cm^{-3}	2.456	2.287
$\lambda(MoK_\alpha)$ / Å	0.71073	0.71073
T / K	150	295
Crystal size / mm^3	0.37 x 0.10 x 0.04	0.44x 0.30 x 0.16
μ / mm^{-1}	6.570	6.488
$F(000)$	544	1464
Refl. collected/unique	2675 / 1882	18811 / 4004
Parameters/restraints	125 / 0	253 / 0
$\Delta\rho_{max}$, $\Delta\rho_{min}$ / e Å^{-3}	1.345; –1.049	0.838; –0.666
$R[F^2 > 4\sigma(F^2)]$	0.0525	0.0544
$wR(F^2)$	0.1364	0.1382
Goodness-of-fit, S	0.930	0.969

Table S1. continued

	(**Clpy**HBr)$_2$(**14tfib**)	(**Clpy**HBr)$_2$(**12tfib**)
Molecular formula	$(C_5H_5NBrCl)_2(C_6F_4I_2)$	$(C_5H_5NBrCl)_2(C_6F_4I_2)$
M_r	790.78	790.78
Crystal system	triclinic	triclinic
Space group	$P\,–1$	$P\,–1$
Crystal data:		
a / Å	4.5389(3)	7.3499(2)
b / Å	7.9487(5)	11.3115(3)
c / Å	16.2280(7)	15.1802(4)
α / °	81.033(5)	109.390(2)
β / °	85.407(4)	94.410(2)
γ / °	83.045(5)	99.943(2)
V / Å^3	572.95(6)	1160.29(6)
Z	1	2
D_{calc} / g cm^{-3}	2.292	2.263
λ(MoK_α) / Å	0.71073	0.71073
T / K	295	295
Crystal size / mm^3	0.40 x 0.31 x 0.14	0.58x 0.38 x 0.05
μ / mm^{-1}	6.502	6.421
F(000)	366	732
Refl. collected/unique	7458 / 2024	23960 / 8071
Parameters/restraints	127 / 0	253 / 0
$\Delta\rho_{max}$, $\Delta\rho_{min}$ / e Å^{-3}	0.429; –0.637	0.854; –0.890
$R[F^2 > 4\sigma(F^2)]$	0.0267	0.0413
w$R(F^2)$	0.0670	0.1052
Goodness-of-fit, S	1.021	0.966

Table S1. continued

	($\mathbf{Brpy}$HBr)$_2$($\mathbf{14tfib}$)	($\mathbf{Brpy}$HBr)($\mathbf{12tfib}$)$_2$
Molecular formula	$(C_5H_5NBr_2)_2(C_6F_4I_2)$	$(C_5H_5NBr_2)(C_6F_4I_2)_2$
M_r	879.70	1042.64
Crystal system	triclinic	orthorombic
Space group	*P* –1	*P bca*
Crystal data:		
a / Å	4.5646(2)	11.8545(10)
b / Å	8.0640(3)	18.5907(16)
c / Å	16.4194(6)	22.698(2)
α / °	79.363(3)	90
β / °	85.269(3)	90
γ / °	82.733(3)	90
V / Å^3	588.14(4)	5002.2(8)
Z	1	8
D_{calc} / g cm^{-3}	2.484	2.769
λ(MoK_α) / Å	0.71073	0.71073
T / K	295	295
Crystal size / mm^3	0.55 x 0.33 x 0.13	0.53 x 0.46 x 0.18
μ / mm^{-1}	9.504	8.244
F(000)	402	3744
Refl. collected/unique	13656 / 2087	41345 / 5454
Parameters/restraints	127 / 0	290 / 0
$\Delta\rho_{max}$, $\Delta\rho_{min}$ / e Å^{-3}	2.236; –2.771	1.444; –1.402
$R[F^2 > 4\sigma(F^2)]$	0.1005	0.0632
w$R(F^2)$	0.2512	0.1739
Goodness-of-fit, S	1.055	1.043

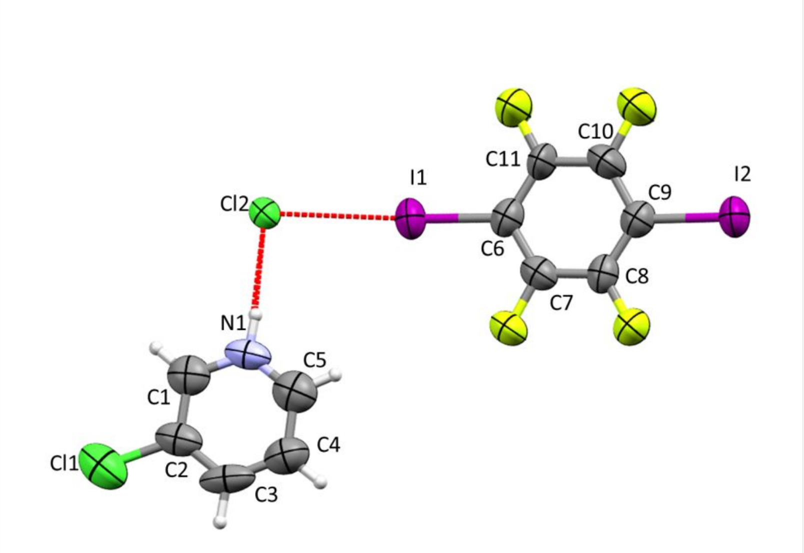

Figure S1. Molecular structure of (**Clpy**HCl)(**14tfib**) showing the atom-labeling scheme. Displacement ellipsoids are drawn at the 50 % probability level, and H atoms are shown as small spheres of arbitrary radius.

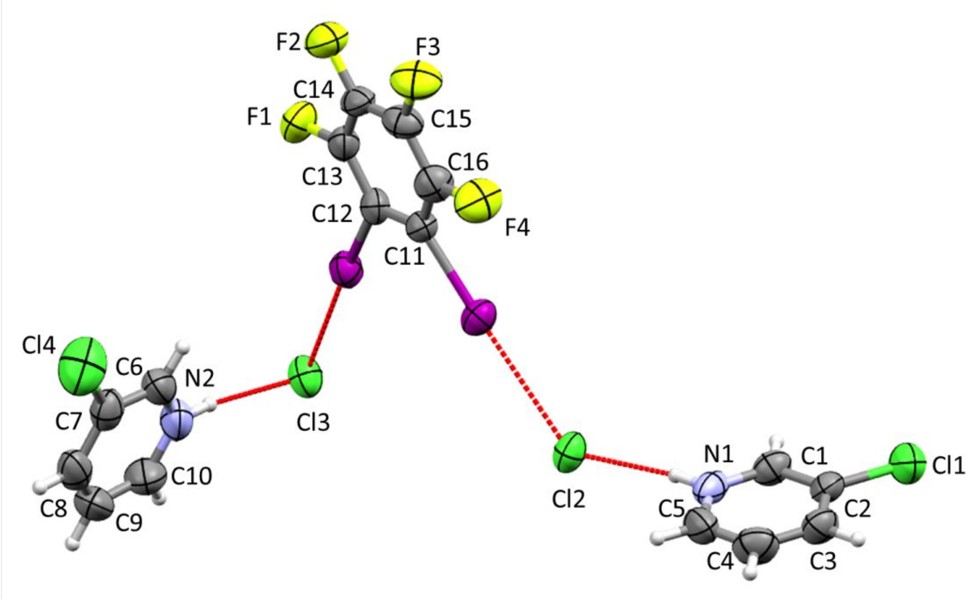

Figure S2. Molecular structure of (**Clpy**HCl)$_2$(**12tfib**) showing the atom-labeling scheme. Displacement ellipsoids are drawn at the 50 % probability level, and H atoms are shown as small spheres of arbitrary radius.

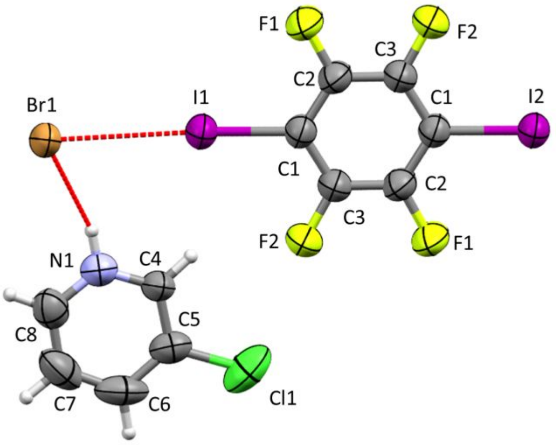

Figure S3. Molecular structure of (**Clpy**HBr)$_2$(**14tfib**) showing the atom-labeling scheme. Displacement ellipsoids are drawn at the 50 % probability level, and H atoms are shown as small spheres of arbitrary radius.

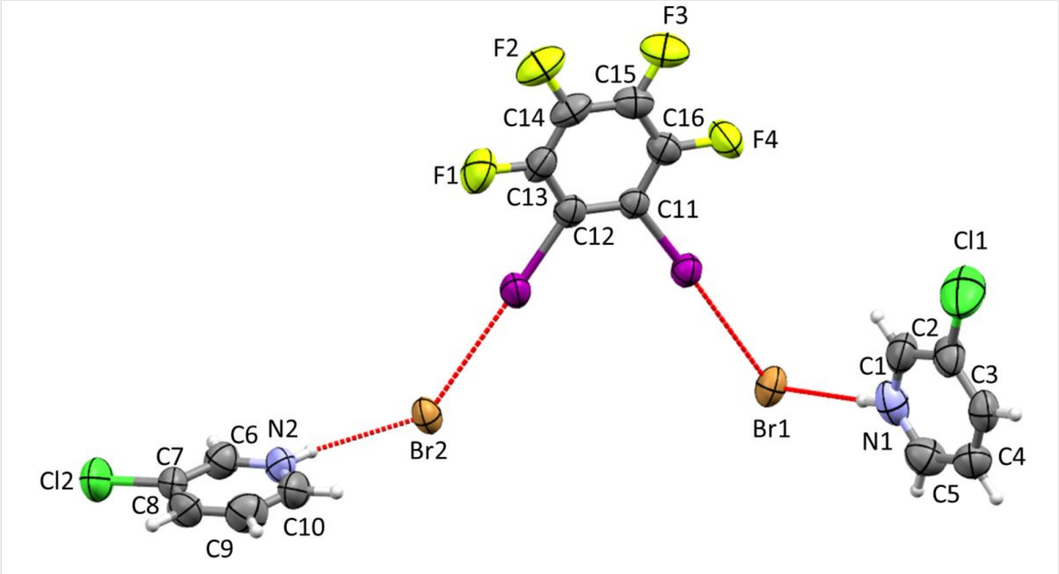

Figure S4. Molecular structure of (**Clpy**HBr)$_2$(**12tfib**) showing the atom-labeling scheme. Displacement ellipsoids are drawn at the 50 % probability level, and H atoms are shown as small spheres of arbitrary radius.

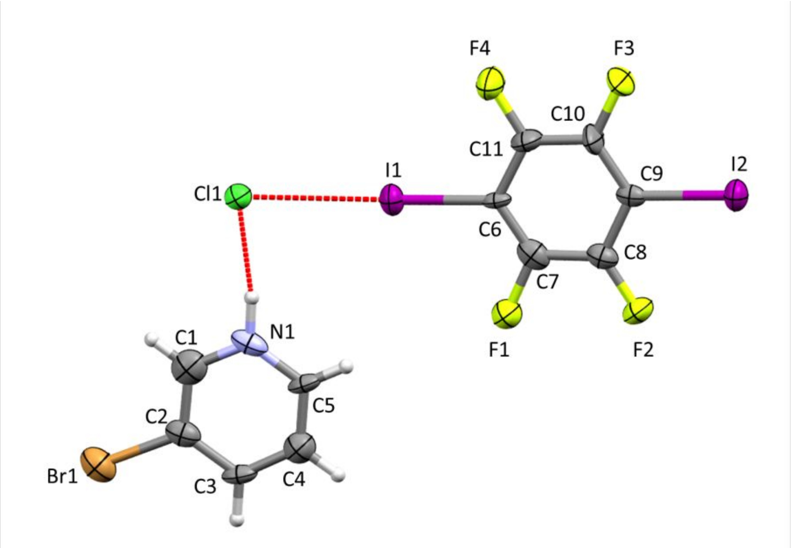

Figure S5. Molecular structure of (**Brpy**HCl)(**14tfib**) showing the atom-labeling scheme. Displacement ellipsoids are drawn at the 50 % probability level, and H atoms are shown as small spheres of arbitrary radius.

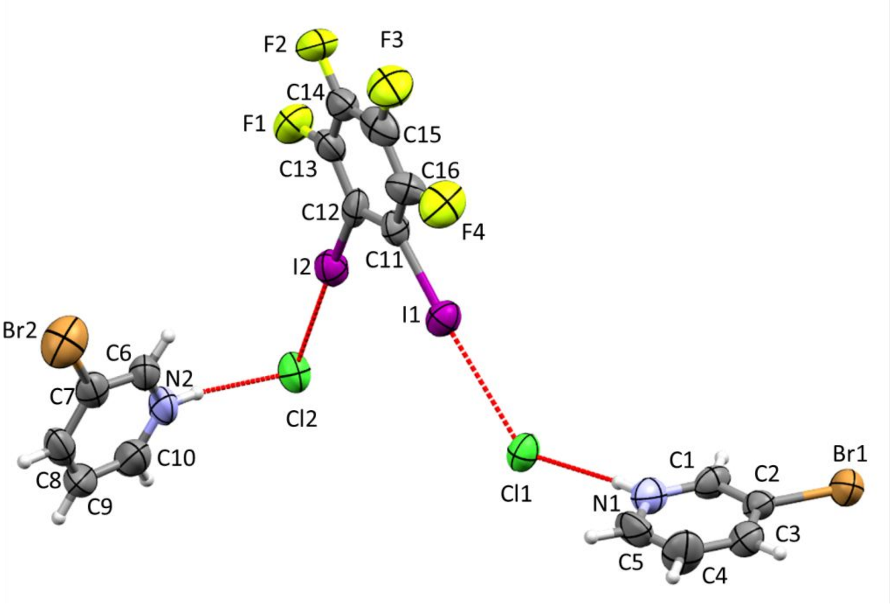

Figure S6. Molecular structure of (**Brpy**HCl)$_2$(**12tfib**) showing the atom-labeling scheme. Displacement ellipsoids are drawn at the 50 % probability level, and H atoms are shown as small spheres of arbitrary radius.

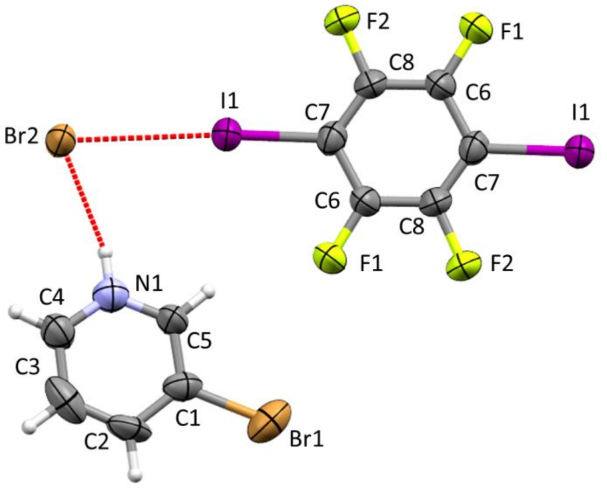

Figure S7. Molecular structure of (**Brpy**HBr)$_2$(**14tfib**) showing the atom-labeling scheme. Displacement ellipsoids are drawn at the 50 % probability level, and H atoms are shown as small spheres of arbitrary radius.

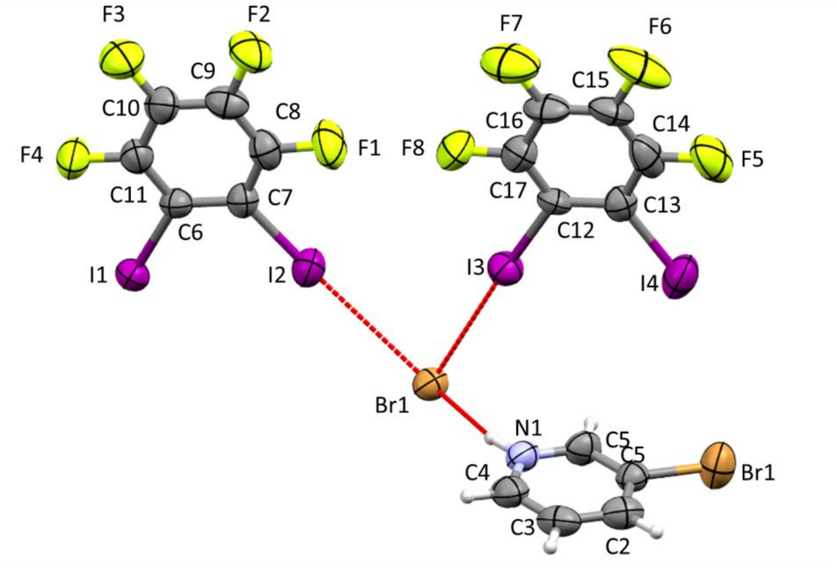

Figure S8. Molecular structure of (**Brpy**HBr)(**12tfib**)$_2$ showing the atom-labeling scheme. Displacement ellipsoids are drawn at the 50 % probability level, and H atoms are shown as small spheres of arbitrary radius.

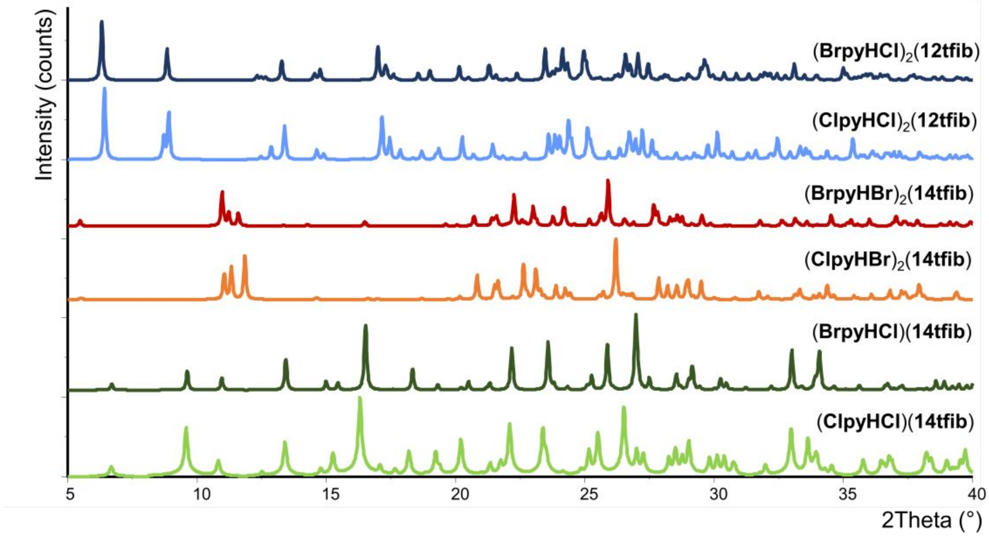

Figure S9. Comparison of powder patterns generated from single crystal data for isostructural cocrystals.

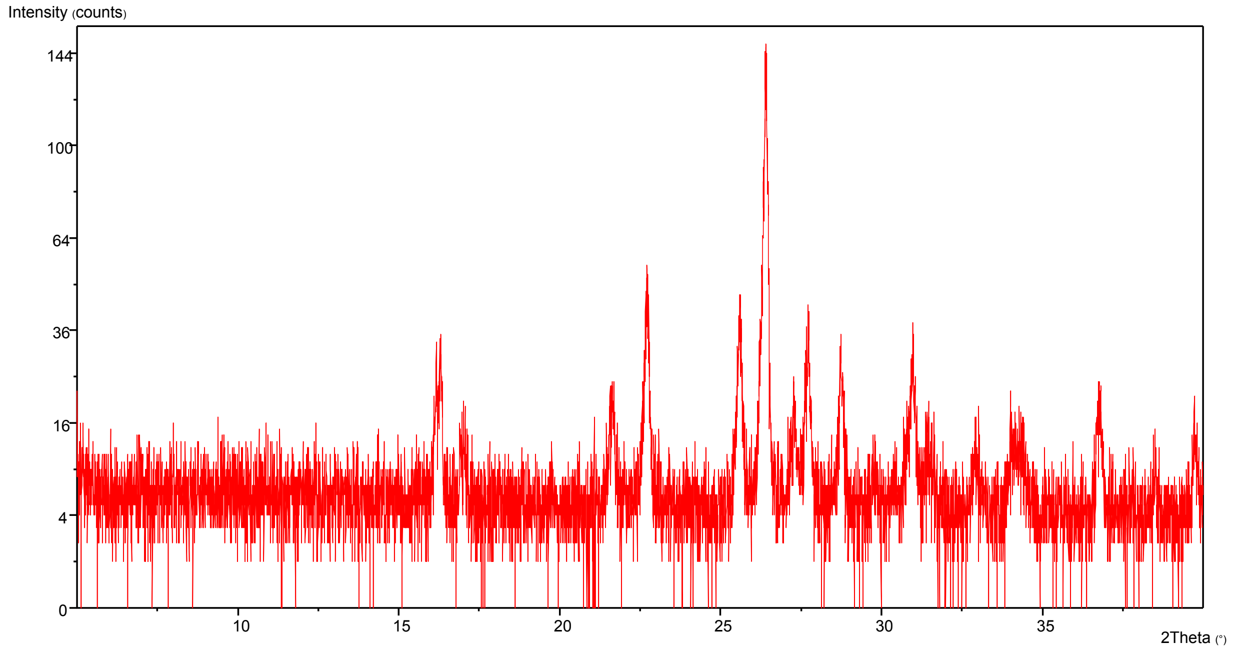

Figure S10. PXRD pattern of **14tfib**.

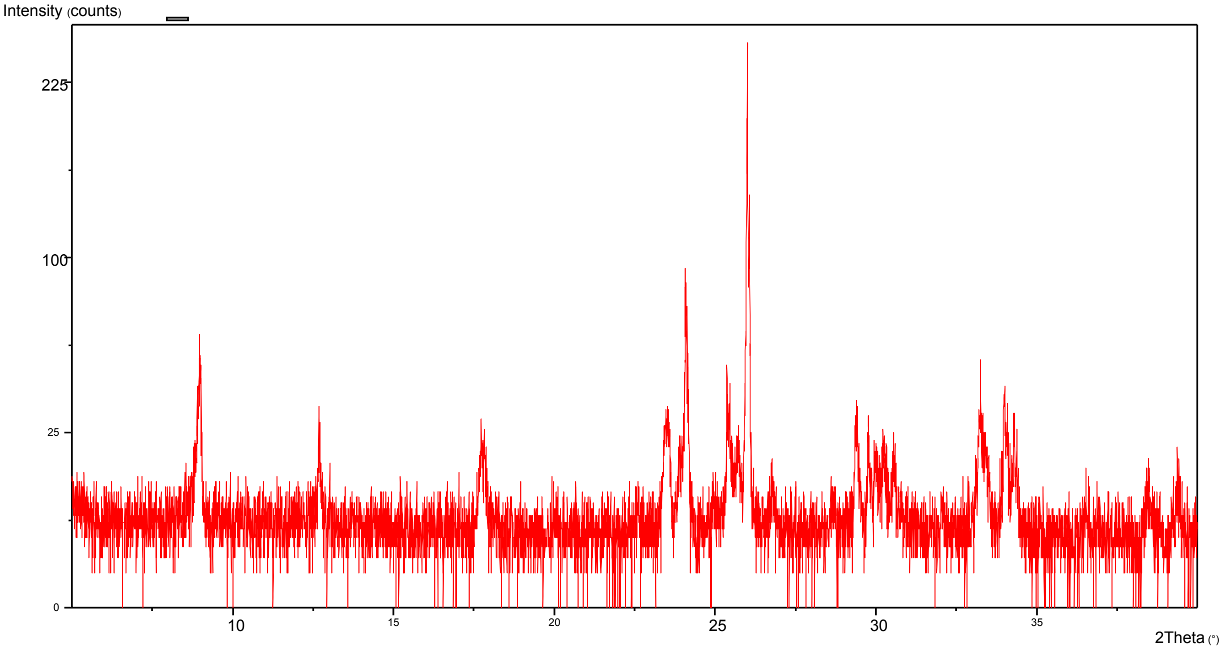

Figure S11. PXRD pattern of **12tfib**.

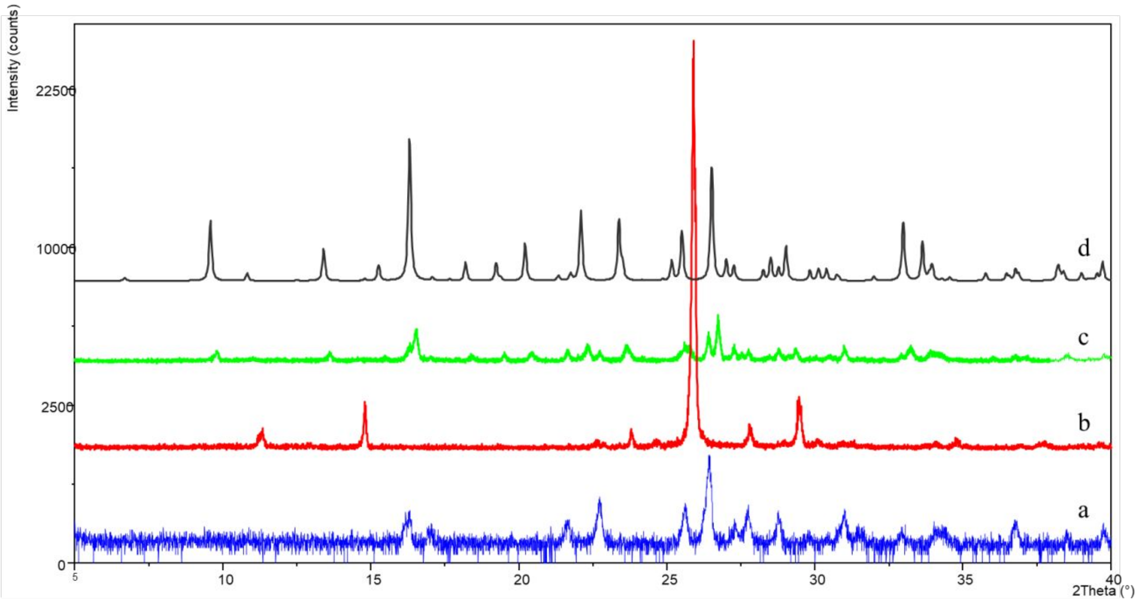

Figure S12. PXRD patterns of: a) **14tfib**, b) **Clpy**HCl, c) product obtained by grinding **14tfib** and **Clpy**HCl in a 1:1 stoichiometric ratio, d) calculated pattern from (**Clpy**HCl)(**14tfib**) single crystal data.

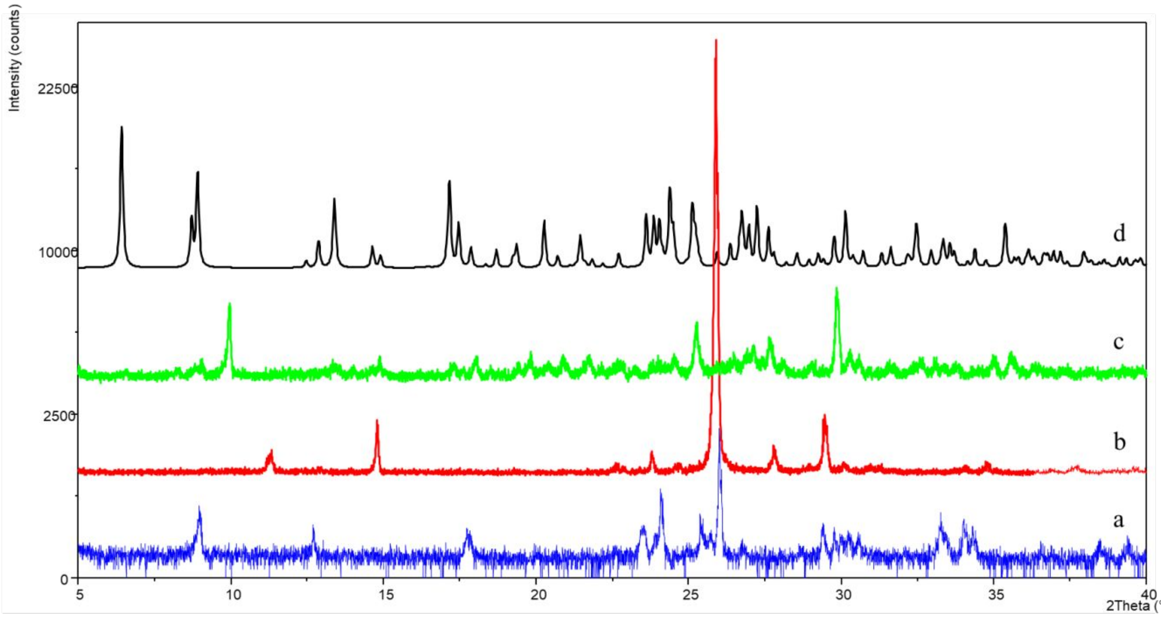

Figure S13. PXRD patterns of: a) **12tfib**, b) **Clpy**HCl, c) product obtained by grinding **12tfib** and **Clpy**HCl in a 1:2 stoichiometric ratio d) calculated pattern from (**Clpy**HCl)$_2$(**12tfib**) single crystal data.

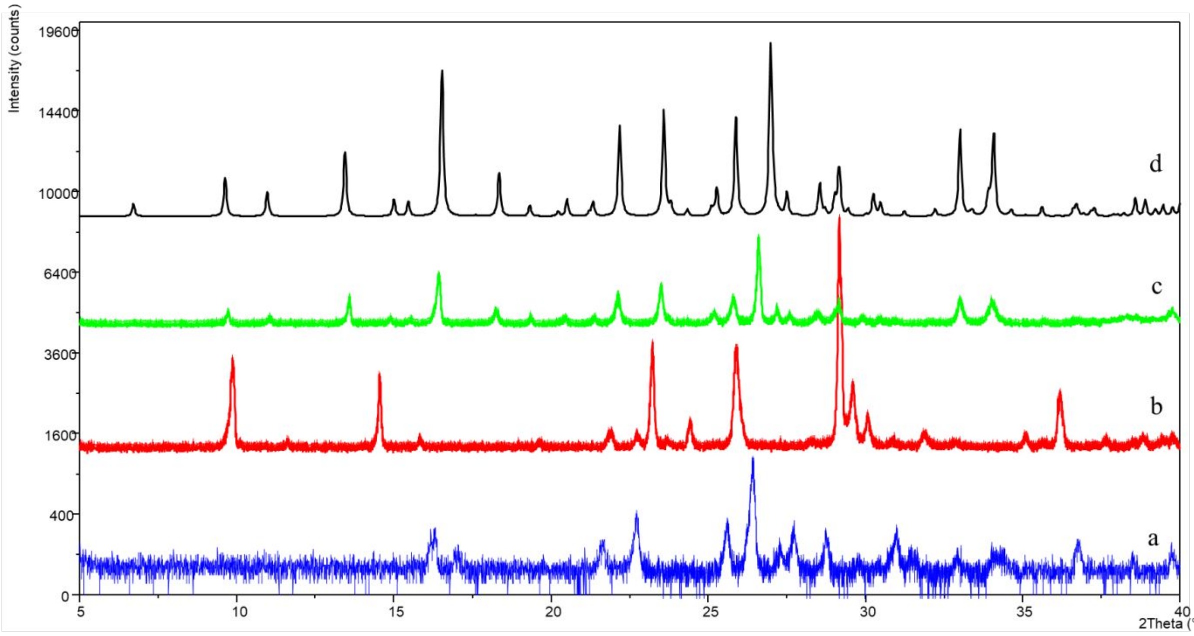

Figure S14. PXRD patterns of: a) **14tfib**, b) **Brpy**HCl, c) product obtained by grinding **14tfib** and **Brpy**HCl in a 1:1 stoichiometric ratio, d) calculated pattern from (**Brpy**HCl)(**14tfib**) single crystal data.

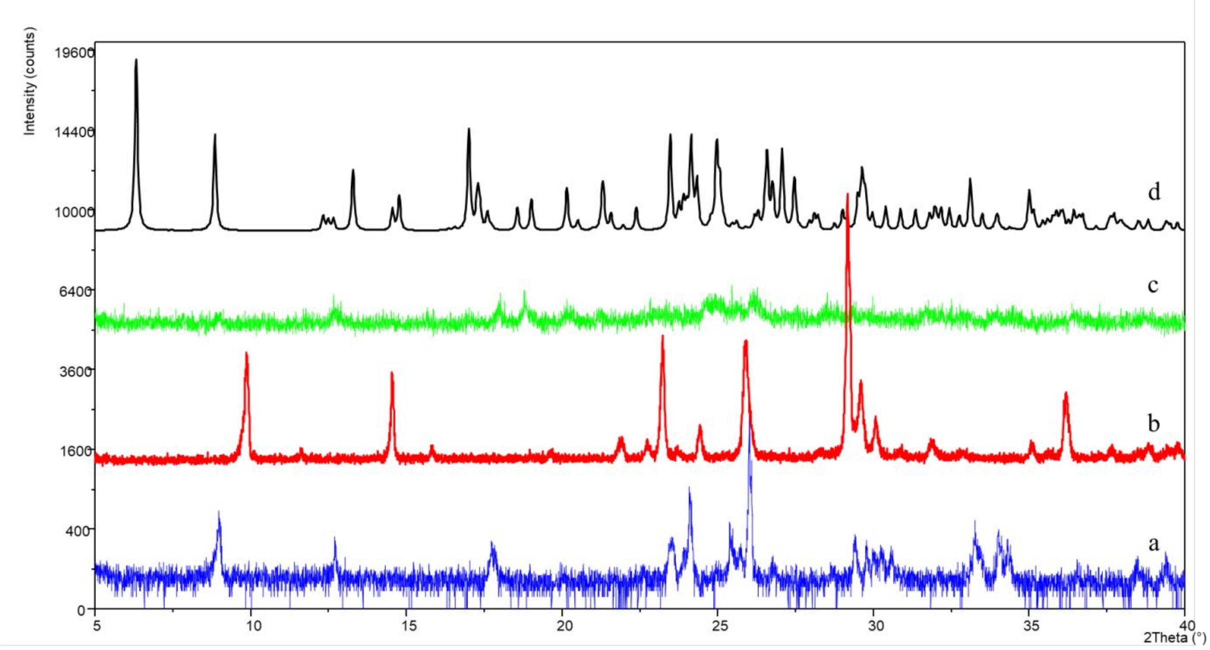

Figure S15. PXRD patterns of: a) **12tfib**, b) **Brpy**HCl, c) product obtained by grinding **12tfib** and **BrpyHCl** in a 1:2 stoichiometric ratio, d) calculated pattern from (**Brpy**HCl)$_2$(**12tfib**) single crystal data.

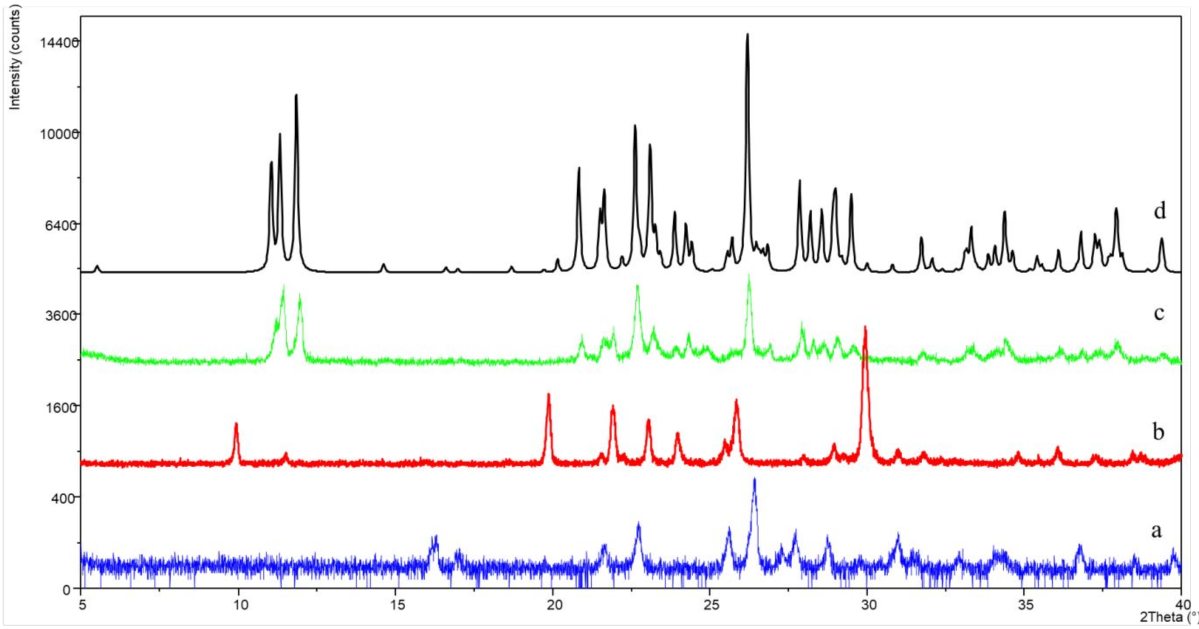

Figure S16. PXRD patterns of: a) **14tfib**, b) **Clpy**HBr, c) product obtained by grinding **14tfib** and **Clpy**HBr in a 1:2 stoichiometric ratio, d) calculated pattern from (**Clpy**HBr)$_2$(**14tfib**) single crystal data.

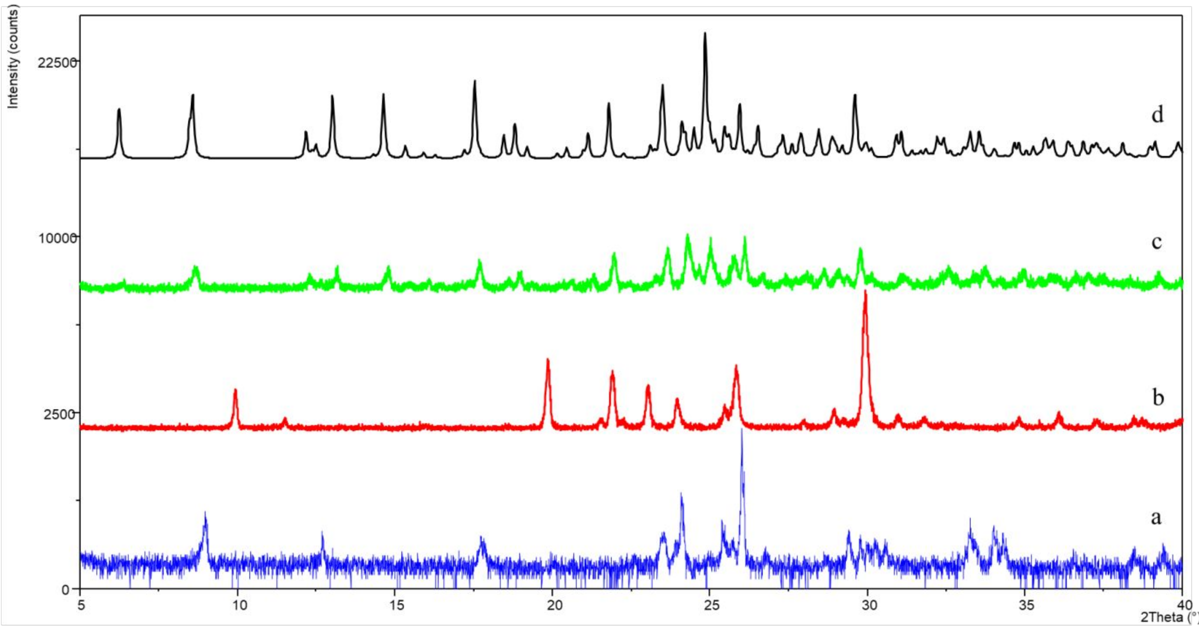

Figure S17. PXRD patterns of: a) **12tfib**, b) **Clpy**HBr, c) product obtained by grinding **12tfib** and **Clpy**HBr in a 1:2 stoichiometric ratio, d) calculated pattern from (**Clpy**HBr)$_2$(**12tfib**) single crystal data.

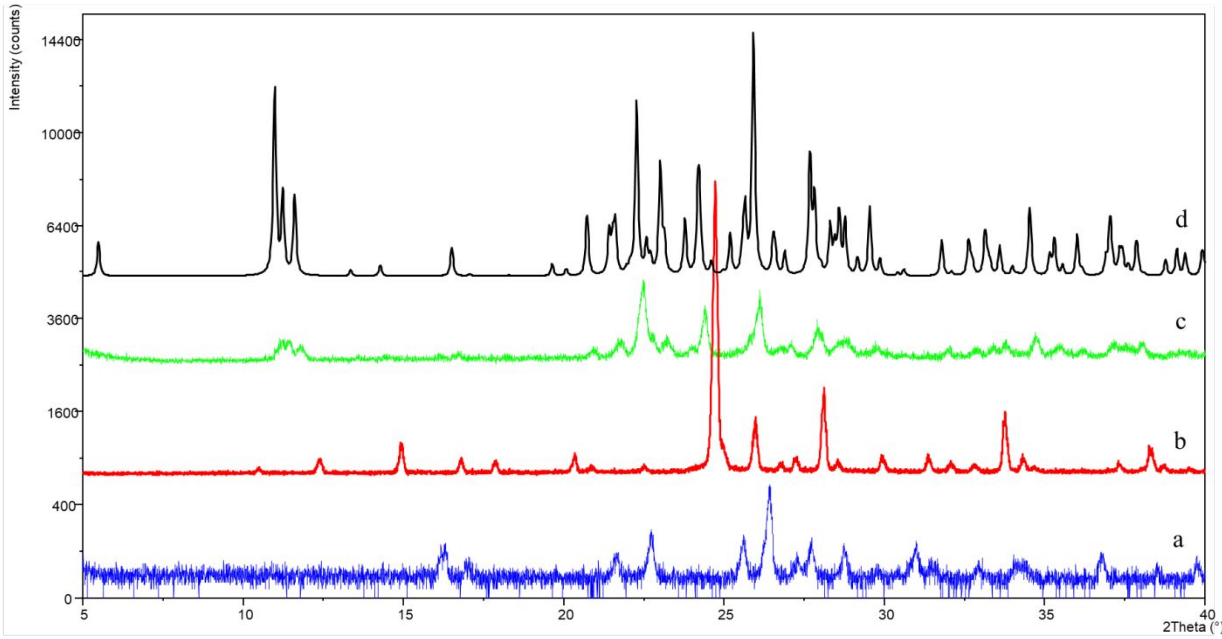

Figure S18. PXRD patterns of: a) **14tfib**, b) **Brpy**HBr, c) product obtained by grinding **14tfib** and **Brpy**HBr in a 1:2 stoichiometric ratio, d) calculated pattern from (**Brpy**HBr)$_2$(**14tfib**) single crystal data.

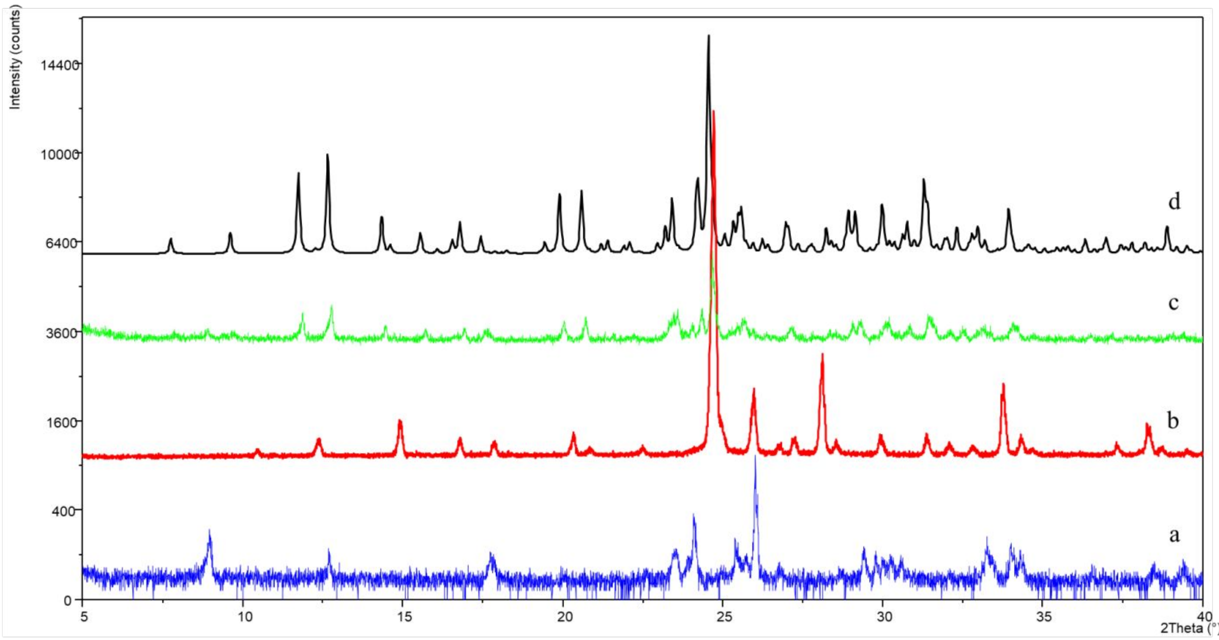

Figure S19. PXRD patterns of: a) **12tfib**, b) **Brpy**HBr, c) product obtained by grinding **12tfib** and **Brpy**HBr in a 2:1 stoichiometric ratio, d) calculated pattern from (**Brpy**HBr)(**12tfib**)$_2$ single crystal data.

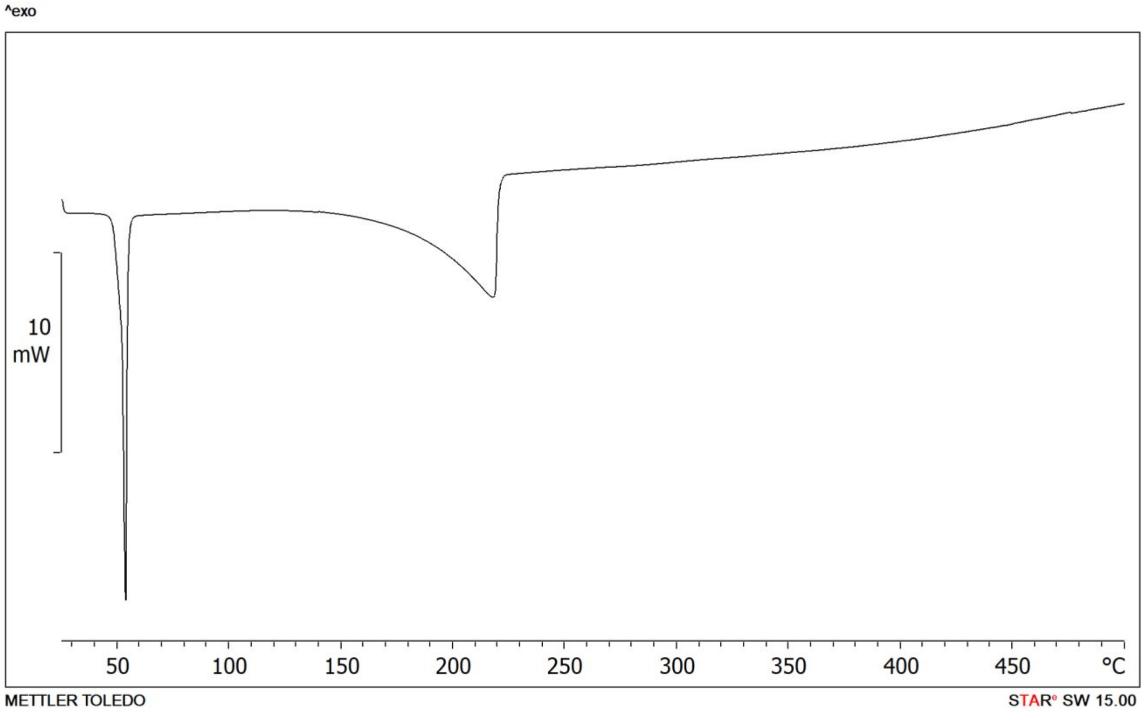

Figure S20. DSC curve of **12tfib**.

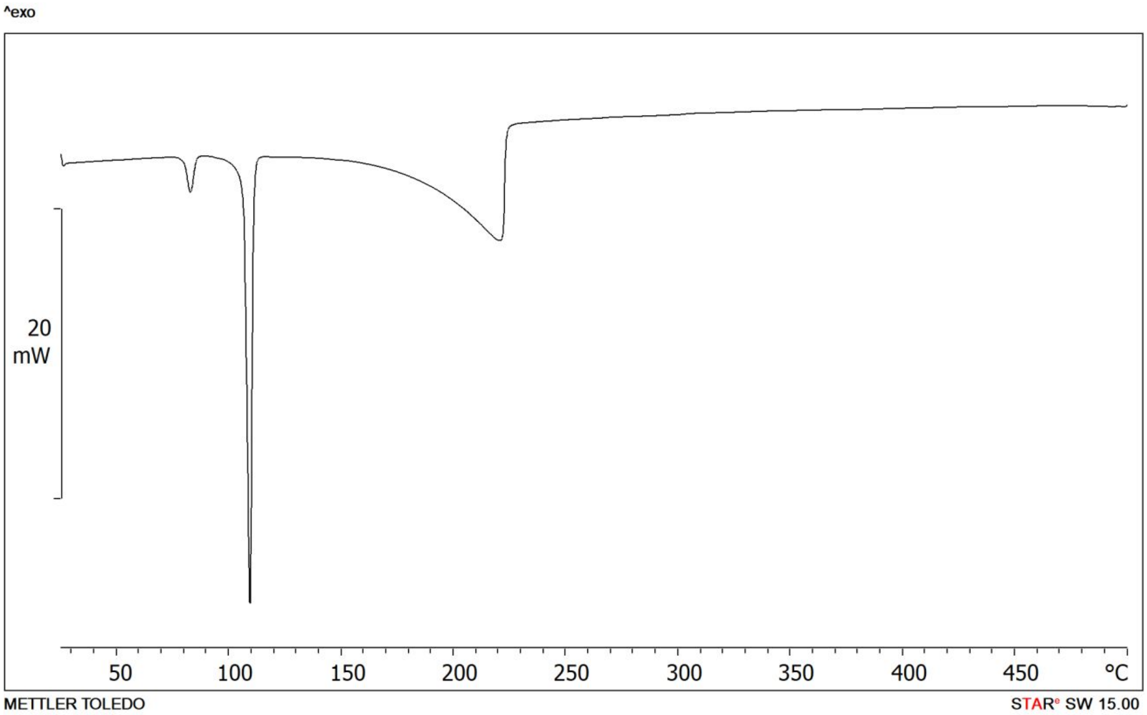

Figure S21. DSC curve of **14tfib**.

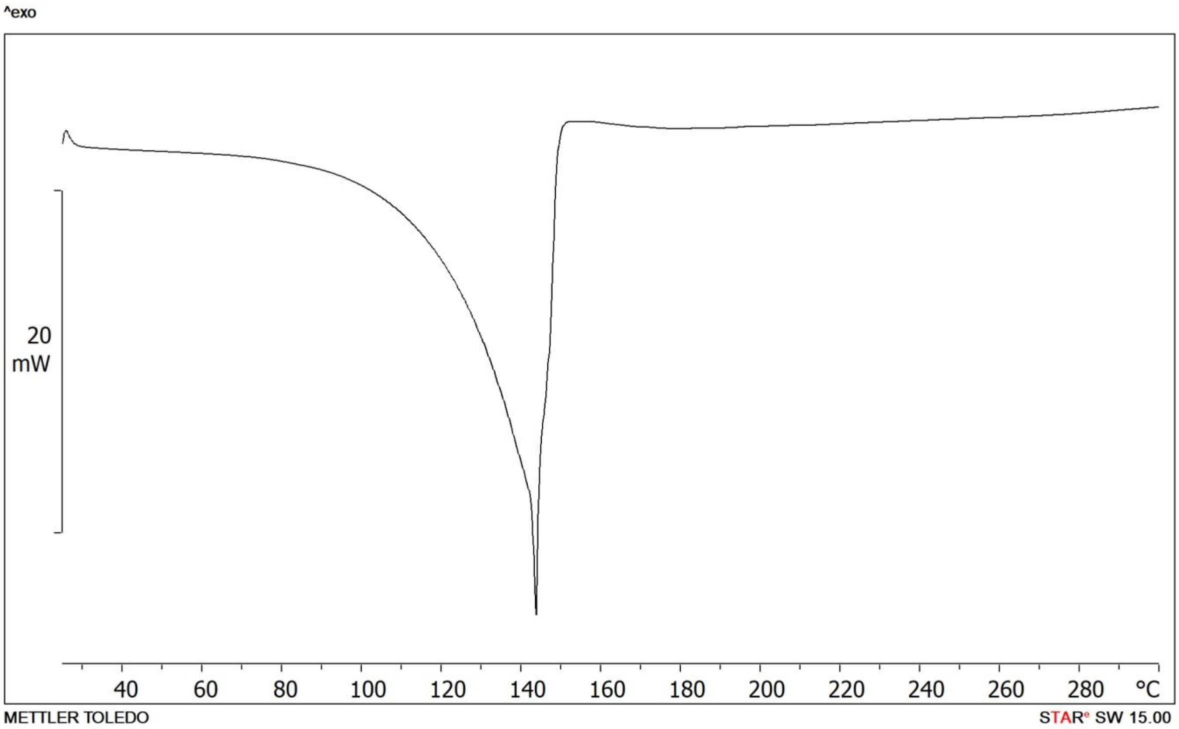

Figure S22. DSC curve of **Clpy**HCl.

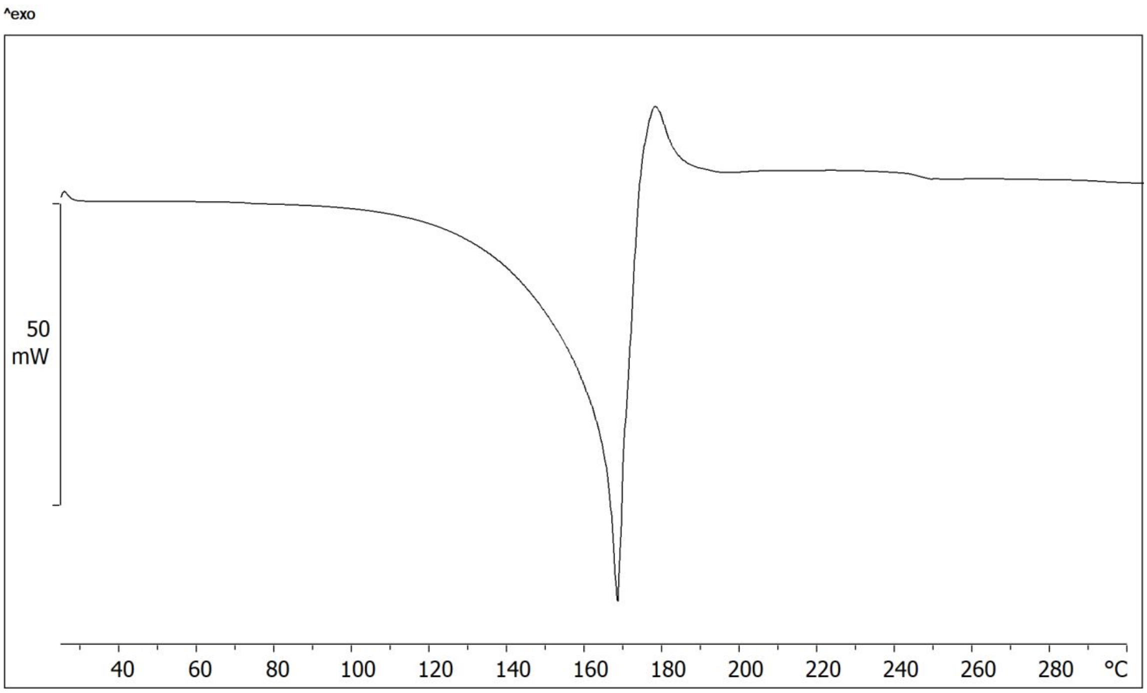

Figure S23. DSC curve of **Brpy**HCl.

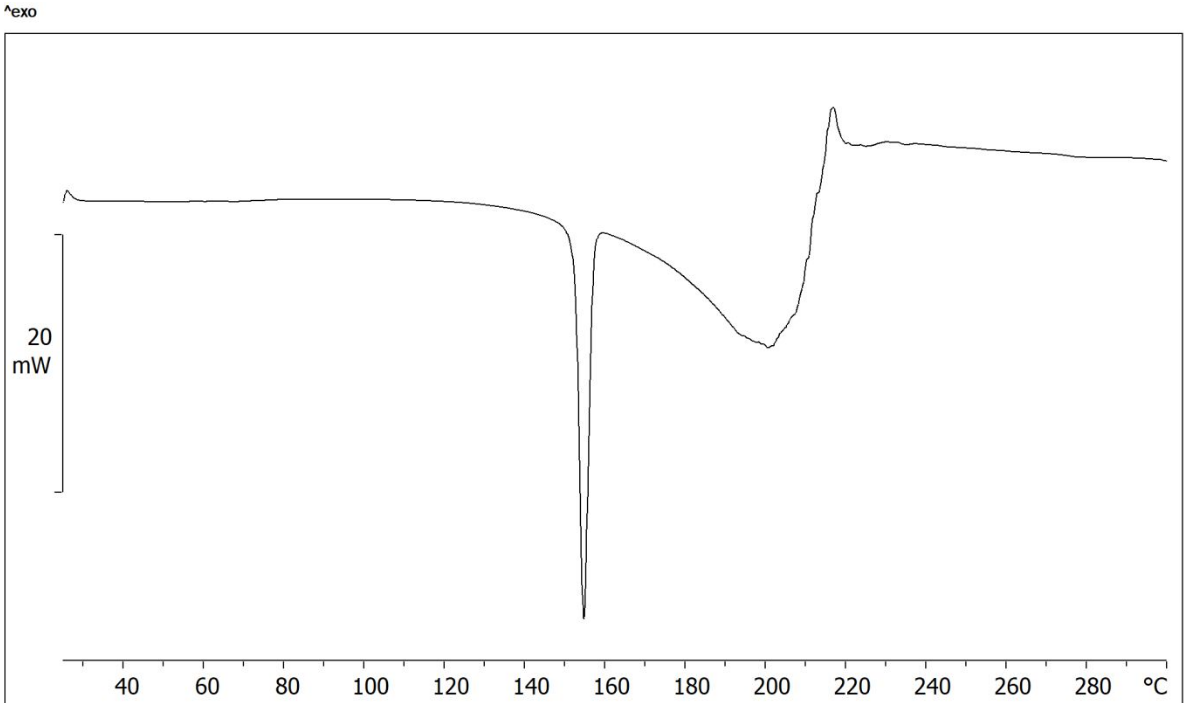

Figure S24. DSC curve of **Clpy**HBr.

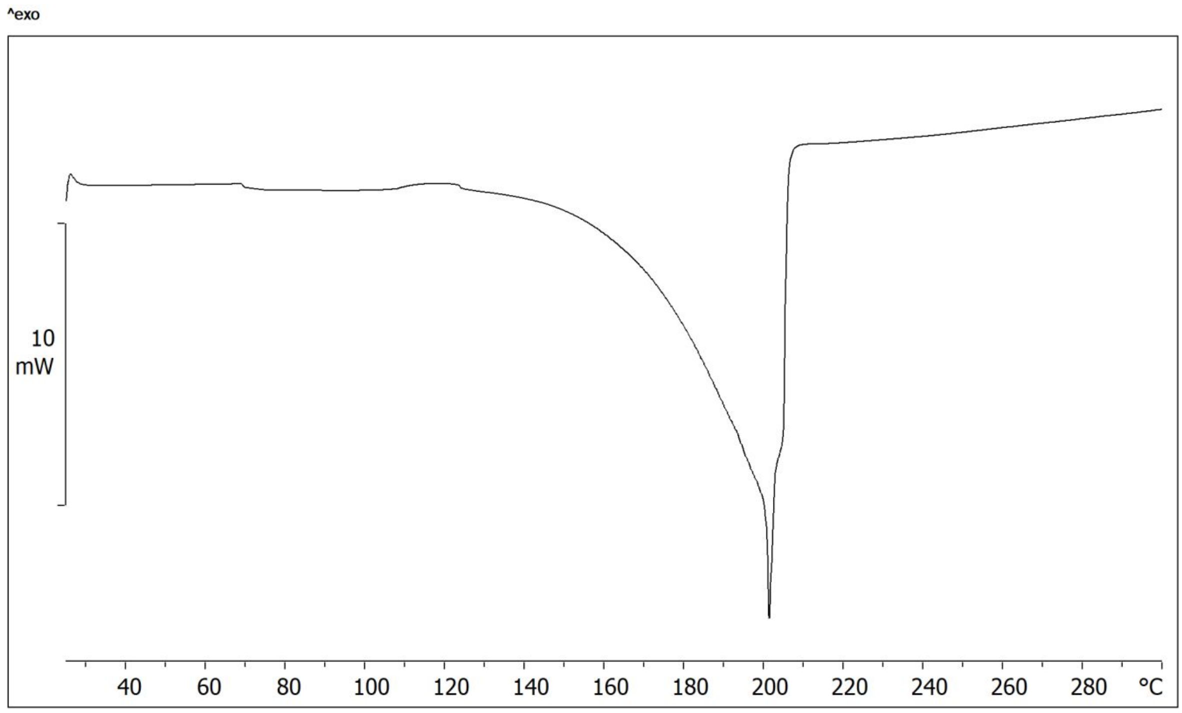

Figure S25. DSC curve of **Brpy**HBr.

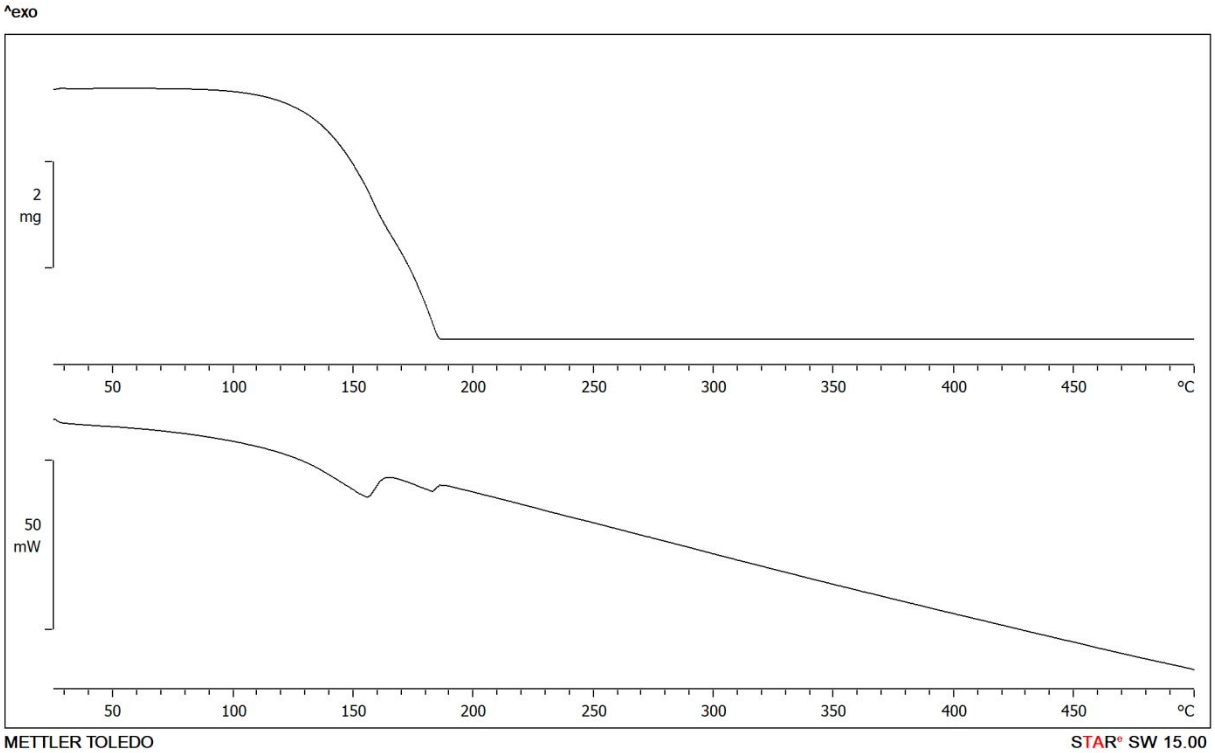

Figure S26. TG and DSC curves of (**Clpy**HCl)(**14tfib**).

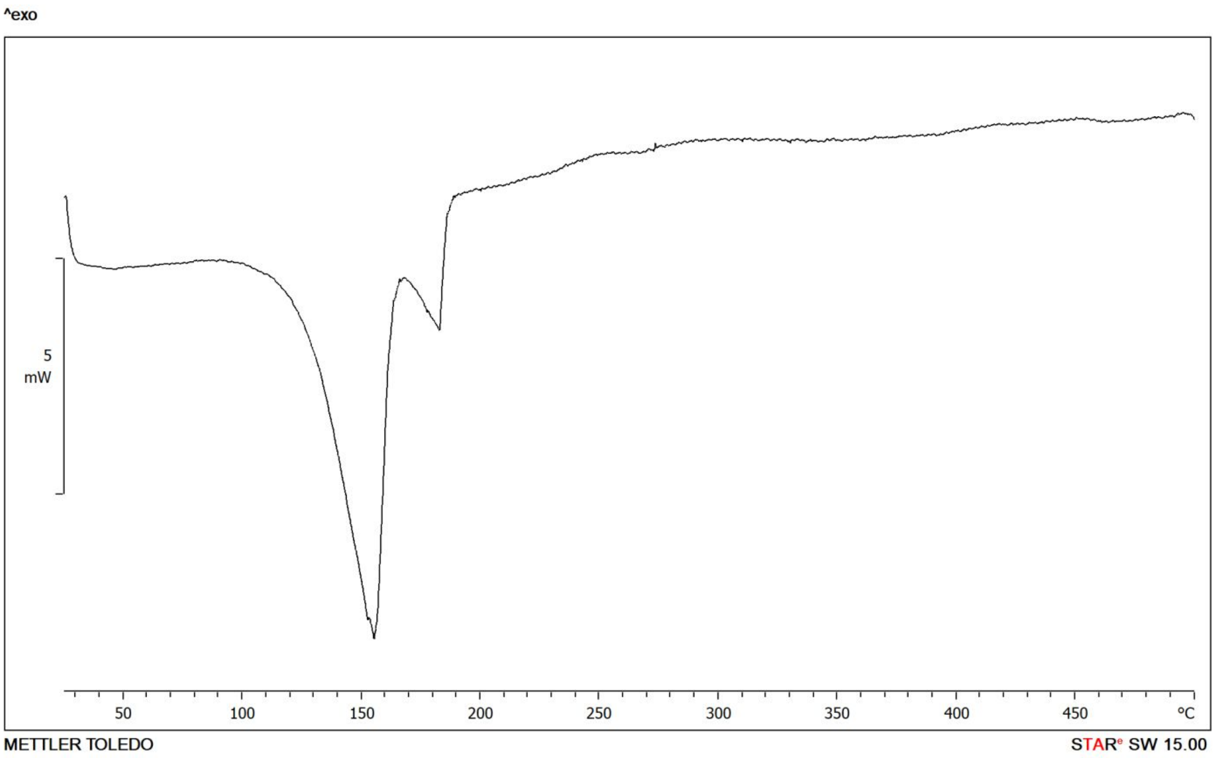

Figure S27. Blank-corrected DSC curve of (**Clpy**HCl)(**14tfib**).

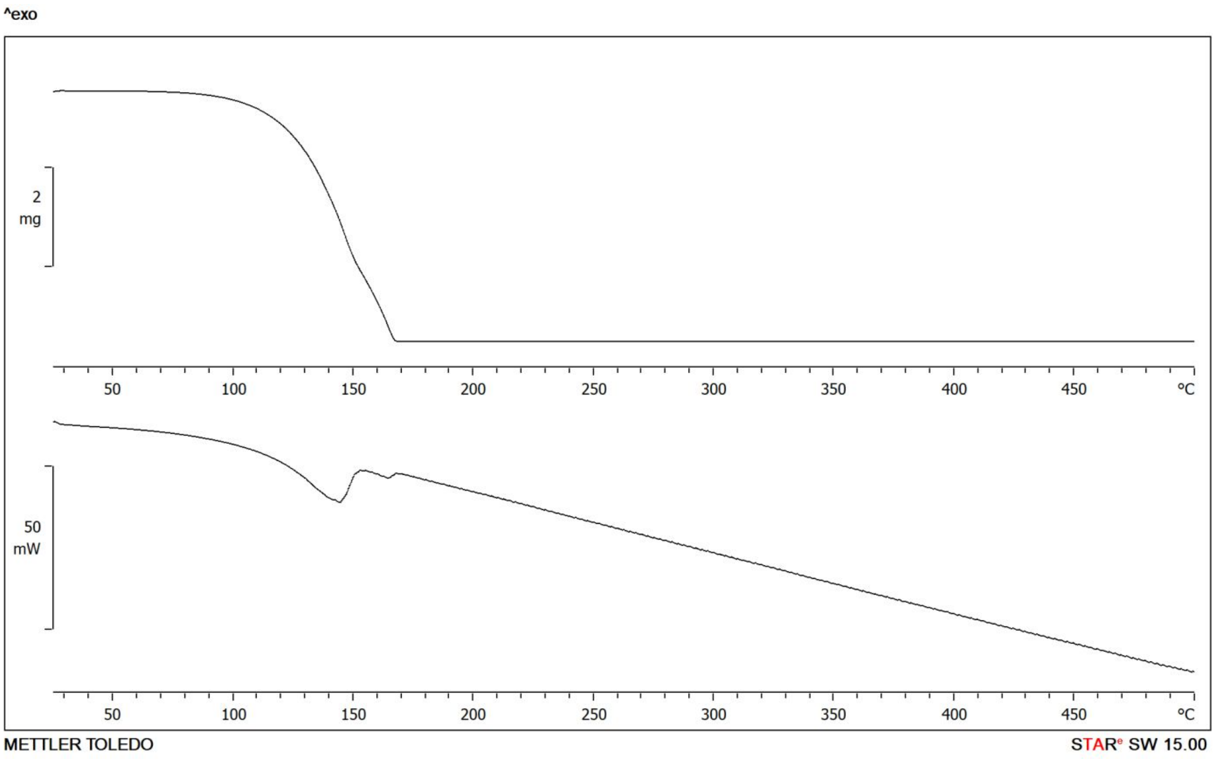

Figure S28. TG and DSC curves of (**Clpy**HCl)$_2$(**12tfib**).

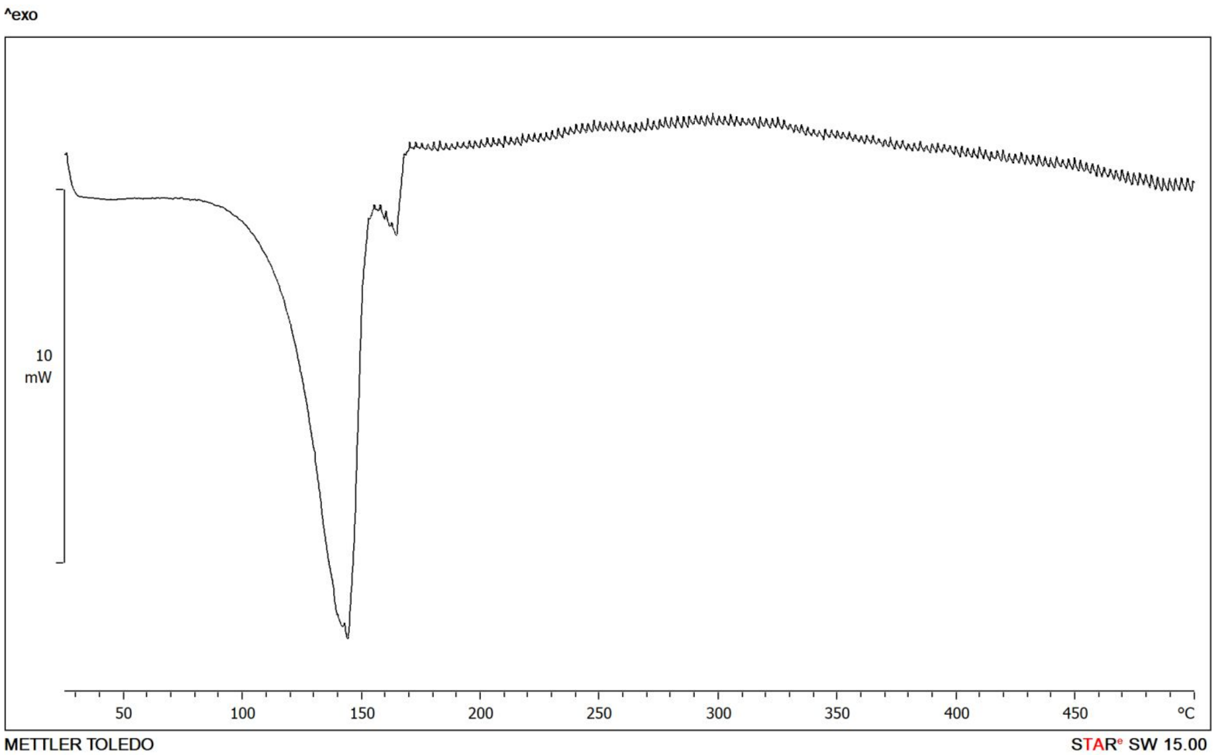

Figure S29. Blank-corrected DSC curve of (**Clpy**HCl)$_2$(**12tfib**).

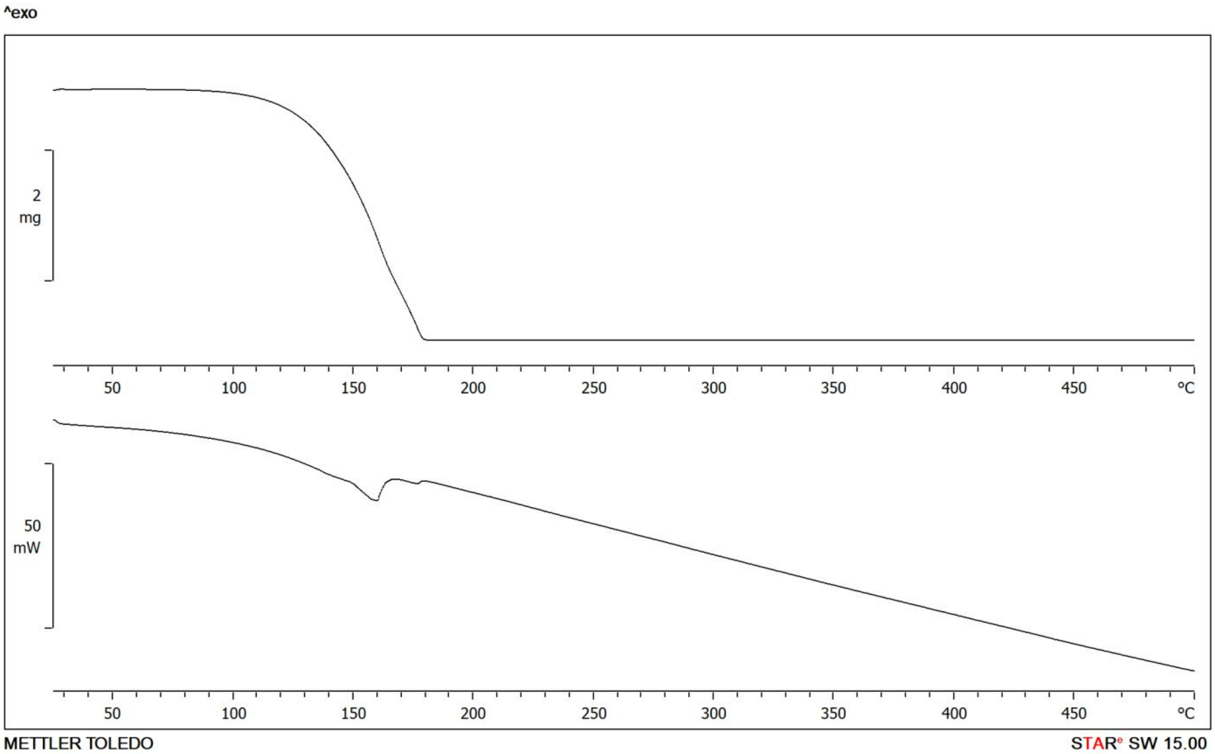

Figure S30. TG and DSC curves of (**Brpy**HCl)(**14tfib**).

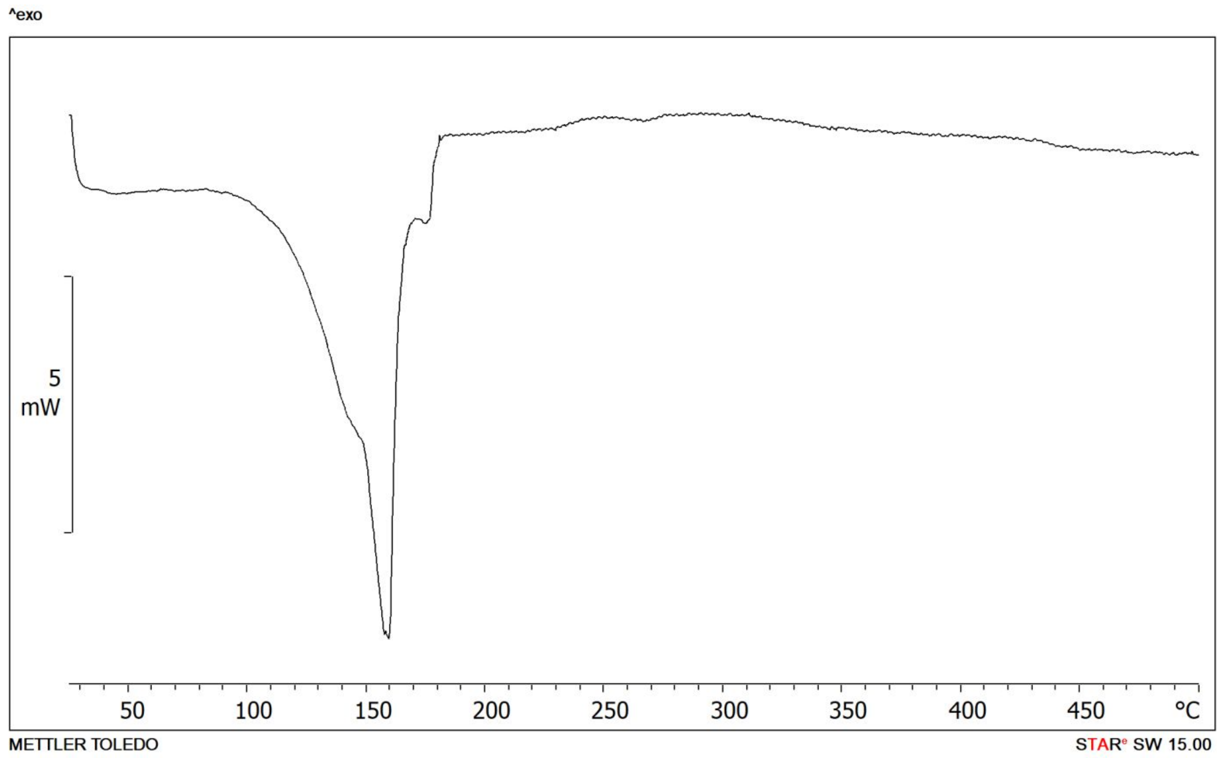

Figure S31. Blank-corrected DSC curve of (**Brpy**HCl)(**14tfib**).

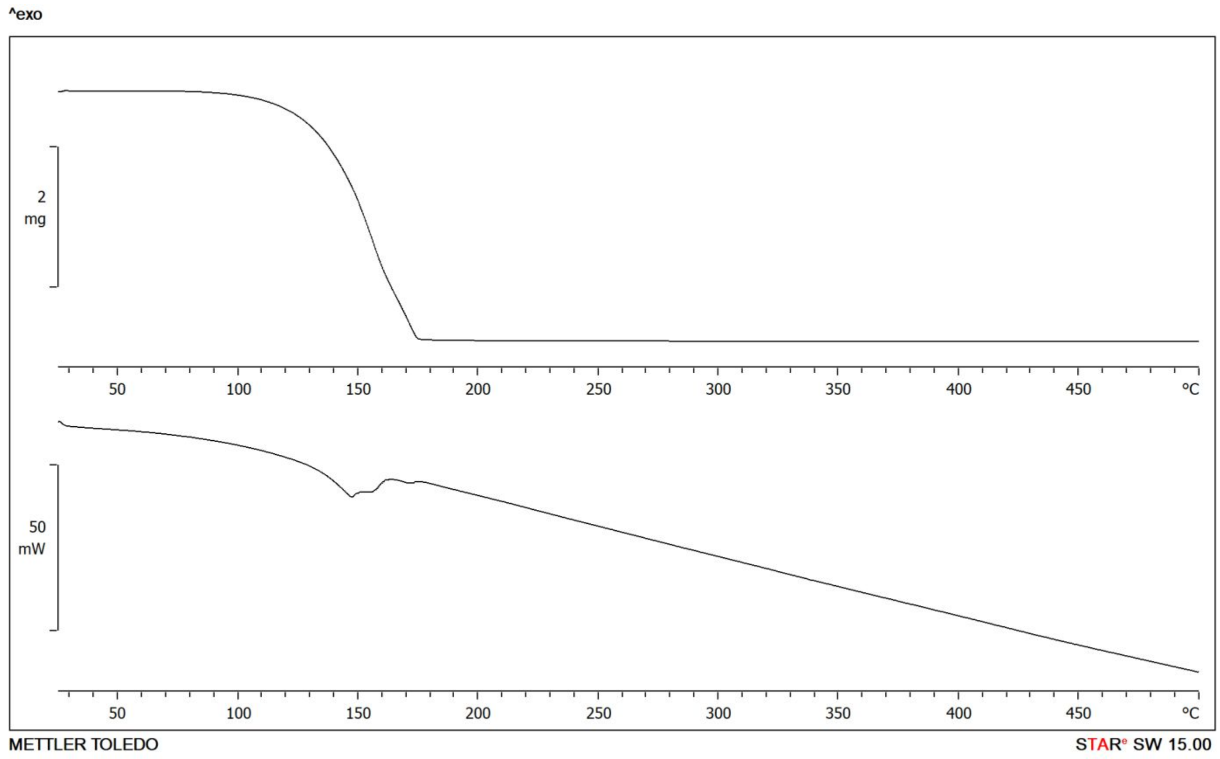

Figure S32. TG and DSC curves of (**Brpy**HCl)$_2$(**12tfib**).

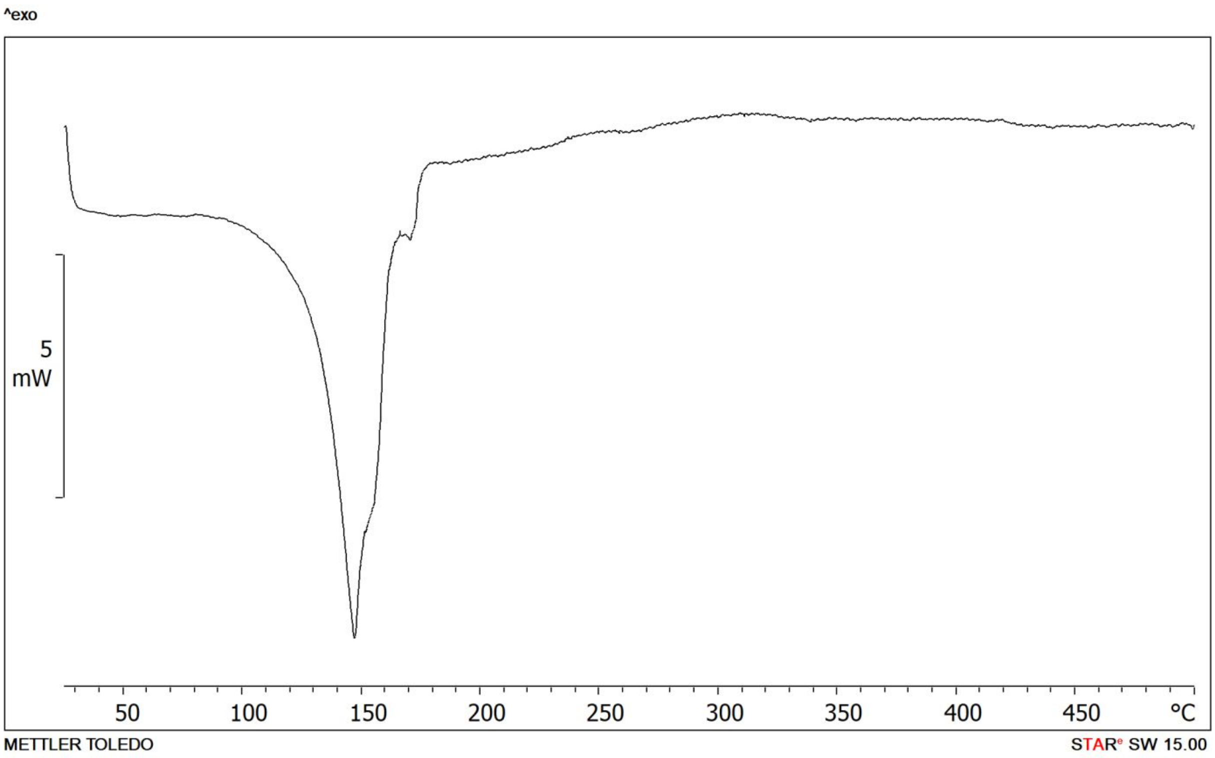

Figure S33. Blank-corrected DSC curve of (**Brpy**HCl)$_2$(**12tfib**).

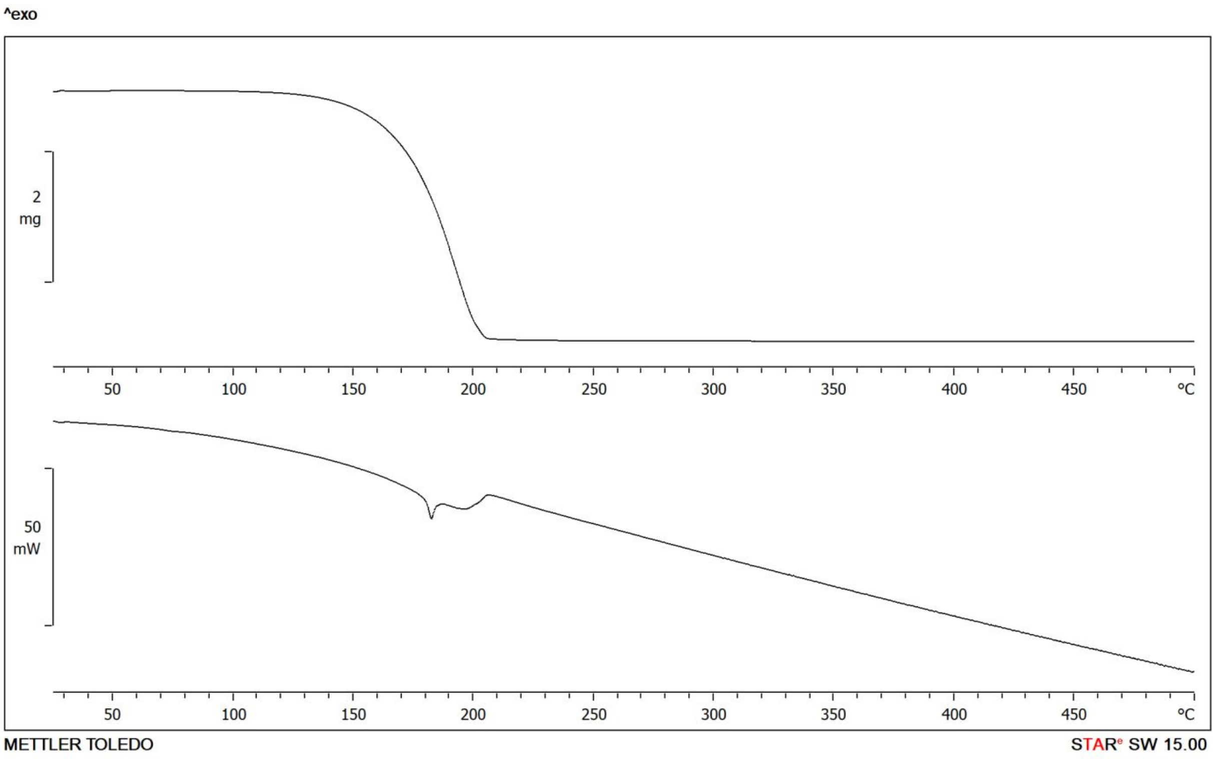

Figure S34. TG and DSC curves of (**Clpy**HBr)$_2$(**14tfib**).

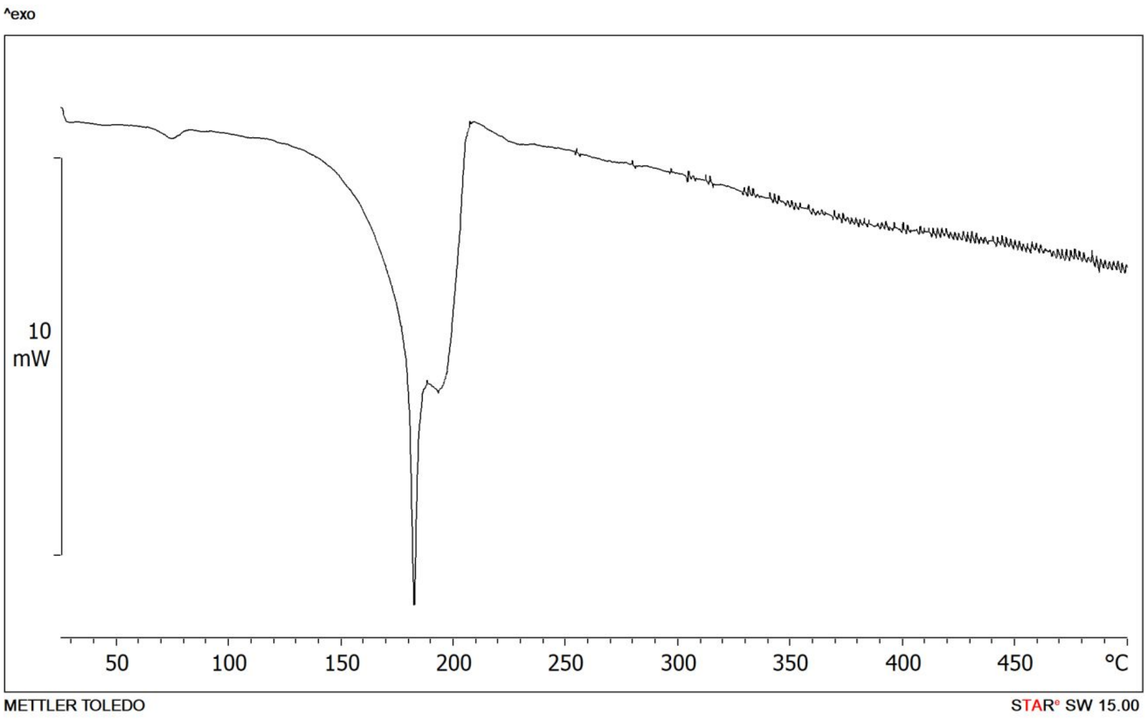

Figure S35. Blank-corrected DSC curve of (**Clpy**HBr)$_2$(**14tfib**).

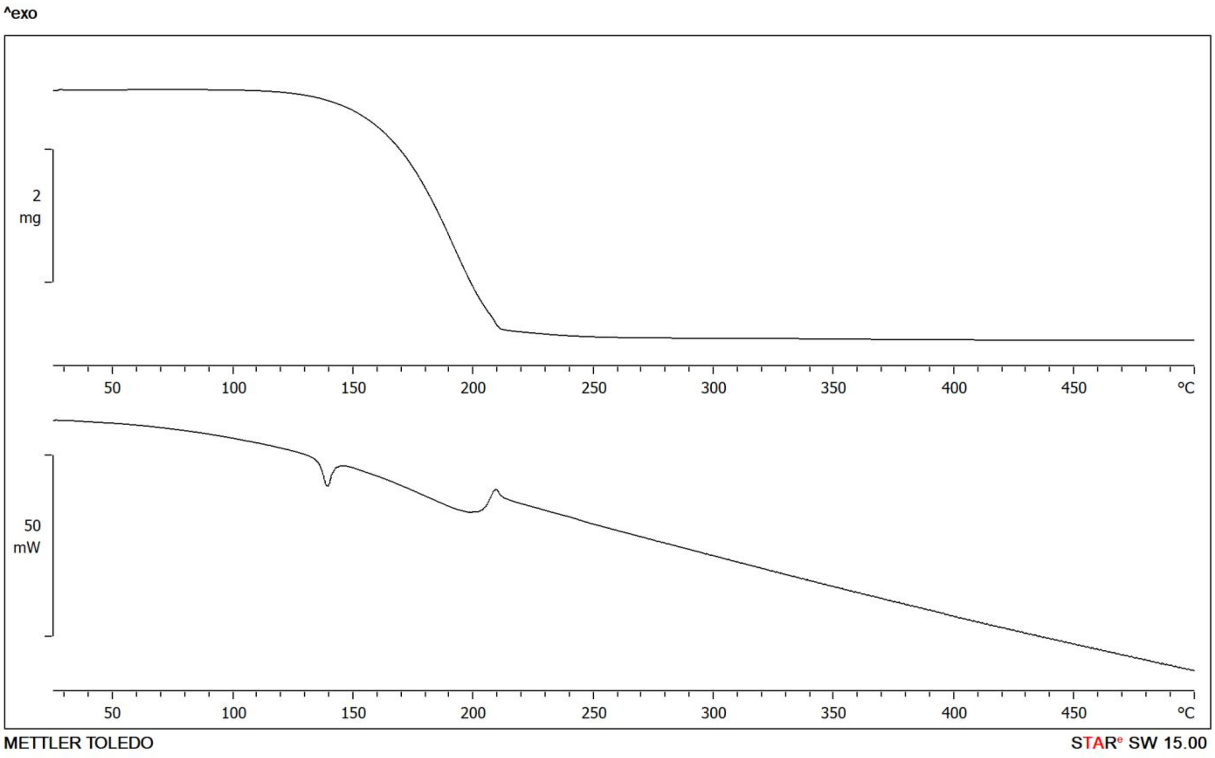

Figure S36. TG and DSC curves of (**Clpy**HBr)$_2$(**12tfib**).

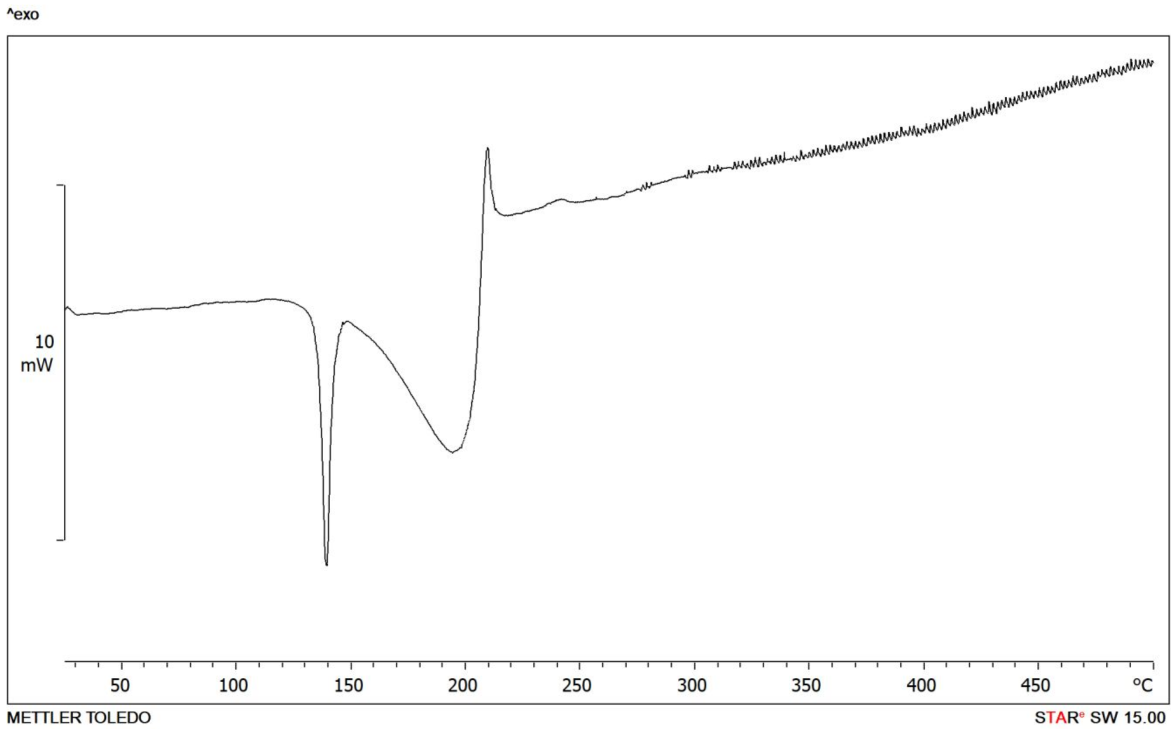

Figure S37. Blank-corrected DSC curve of (**Clpy**HBr)$_2$(**12tfib**).

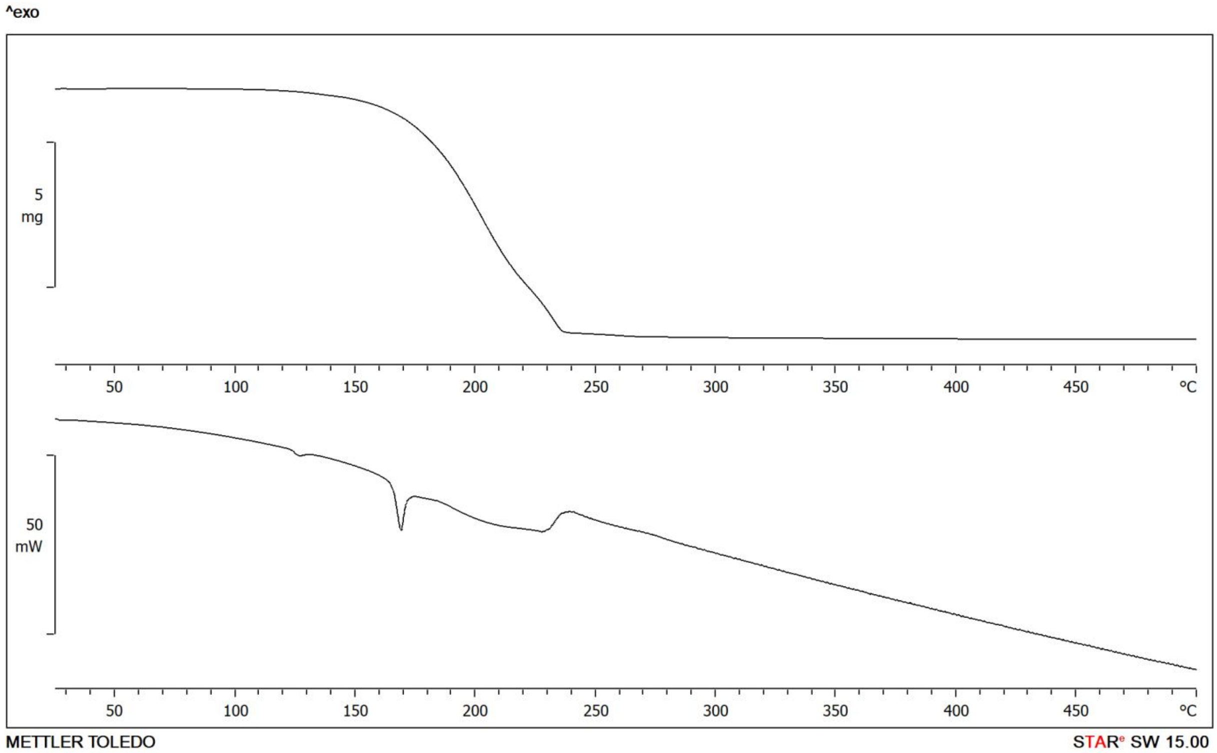

Figure S38. TG and DSC curves of (**Brpy**HBr)$_2$(**14tfib**).

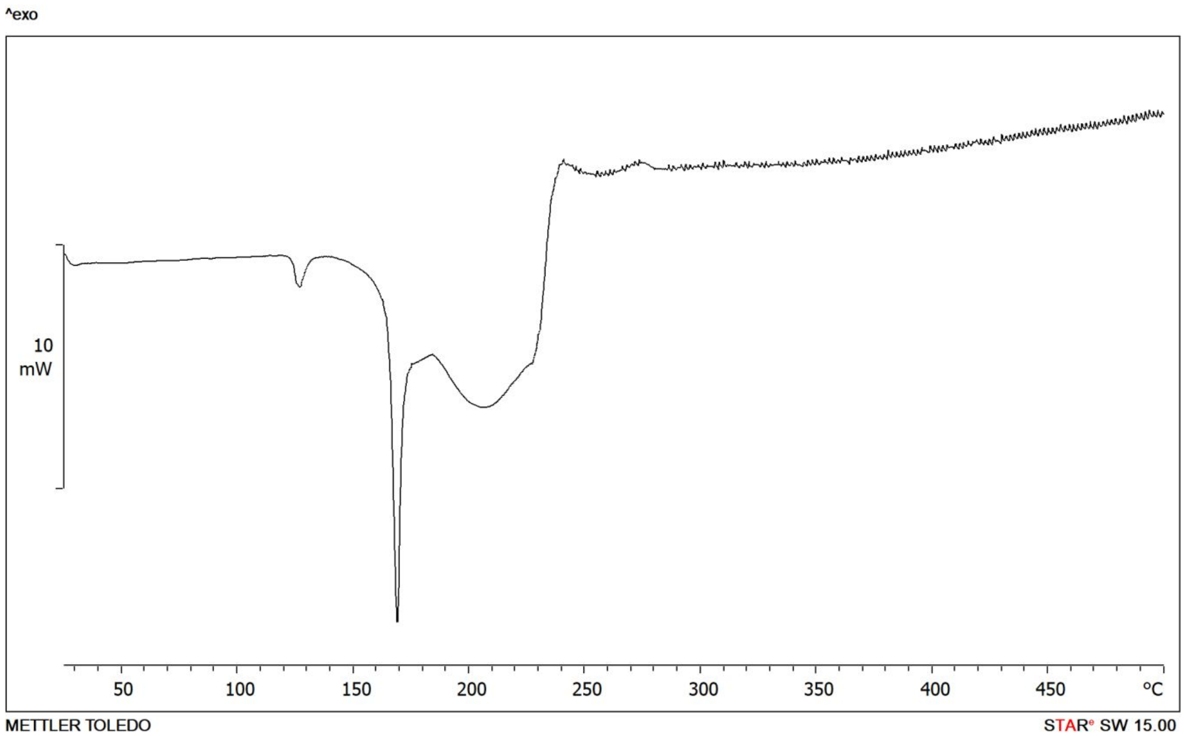

Figure S39. Blank-corrected DSC curve of (**Brpy**HBr)$_2$(**14tfib**).

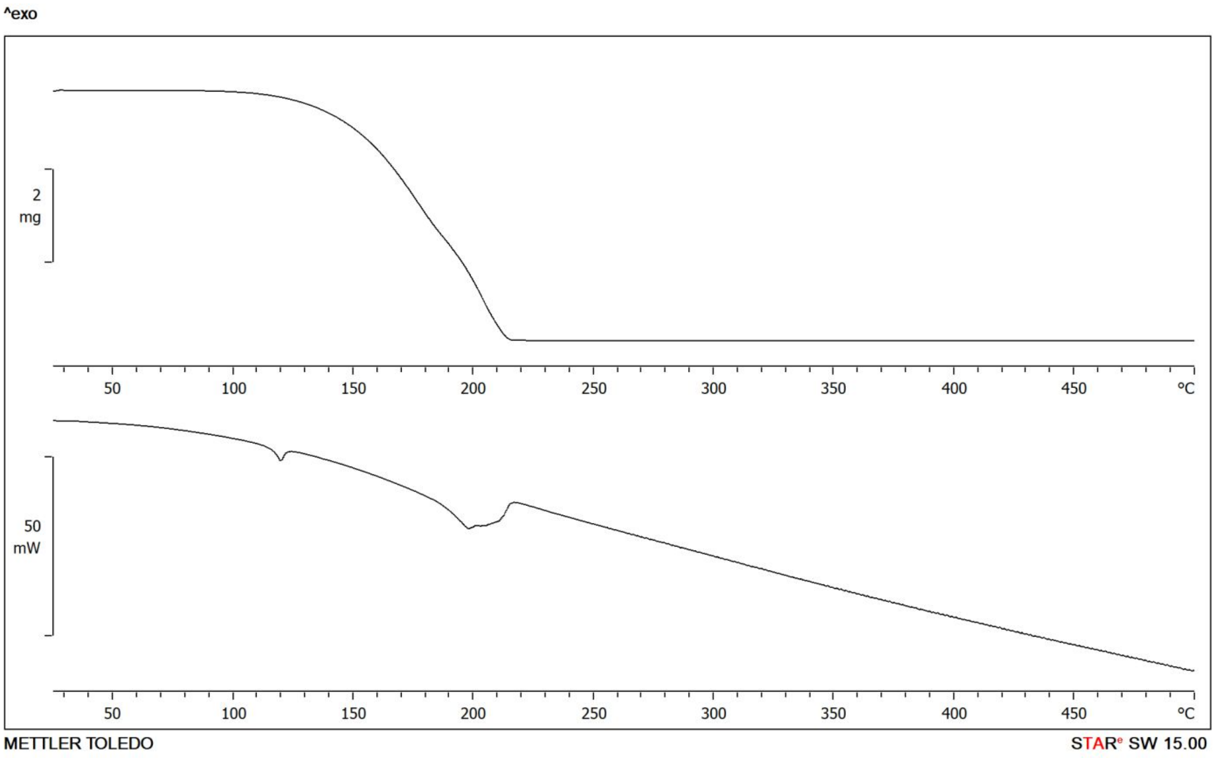

Figure S40. TG and DSC curves of (**Brpy**HBr)(**12tfib**)$_2$.

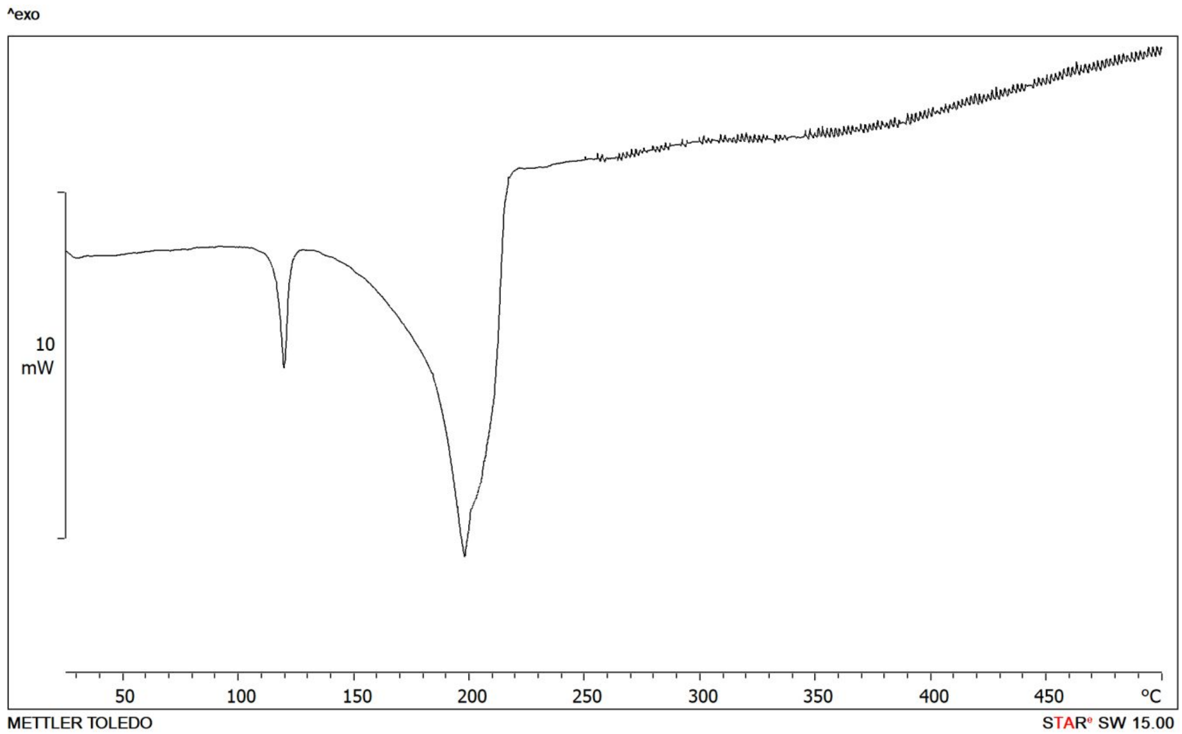

Figure S41. Blank-corrected DSC curve of (**Brpy**HBr)(**12tfib**)$_2$.
